# Association of MAFLD and MASLD with all-cause and cause-specific dementia: a prospective cohort study

**DOI:** 10.1186/s13195-024-01498-5

**Published:** 2024-06-26

**Authors:** Xue Bao, Lina Kang, Songjiang Yin, Gunnar Engström, Lian Wang, Wei Xu, Biao Xu, Xiaowen Zhang, Xinlin Zhang

**Affiliations:** 1 Department of Cardiology, Nanjing Drum Tower Hospital, the Affiliated Hospital of Nanjing University Medical School, 321 Zhongshan Road, Nanjing, 210008 China; 2 Department of Clinical Sciences, Lund University, Malmö, Sweden; 3 Departments of Orthopedics, Jiangsu Province Hospital of Chinese Medicine, the Affiliated Hospital of Nanjing University of Chinese Medicine, Nanjing, China; 4 Department of Endocrinology, Nanjing Drum Tower Hospital, the Affiliated Hospital of Nanjing University Medical School, Nanjing, China; 5 Endocrine and Metabolic Disease Medical Center, Nanjing Drum Tower Hospital, the Affiliated Hospital of Nanjing University Medical School, Nanjing, China

**Keywords:** Liver disease, MAFLD, MASLD, Dementia, Prospective, UK Biobank

## Abstract

**Background:**

Liver disease and dementia are both highly prevalent and share common pathological mechanisms. We aimed to investigate the associations between metabolic dysfunction-associated fatty liver disease (MAFLD), metabolic dysfunction-associated steatotic liver disease (MASLD) and the risk of all-cause and cause-specific dementia.

**Methods:**

We conducted a prospective study with 403,506 participants from the UK Biobank. Outcomes included all-cause dementia, Alzheimer’s disease, and vascular dementia. Multivariable Cox proportional hazards models were used for analyses.

**Results:**

155,068 (38.4%) participants had MAFLD, and 111,938 (27.7%) had MASLD at baseline. During a median follow-up of 13.7 years, 5,732 participants developed dementia (2,355 Alzheimer’s disease and 1,274 vascular dementia). MAFLD was associated with an increased risk of vascular dementia (HR 1.32 [95% CI 1.18–1.48]) but a reduced risk of Alzheimer’s disease (0.92 [0.84–1.0]). Differing risks emerged among MAFLD subtypes, with the diabetes subtype increasing risk of all-cause dementia (1.8 [1.65–1.96]), vascular dementia (2.95 [2.53–3.45]) and Alzheimer’s disease (1.46 [1.26–1.69]), the lean metabolic disorder subtype only increasing vascular dementia risk (2.01 [1.25–3.22]), whereas the overweight/obesity subtype decreasing risk of Alzheimer’s disease (0.83 [0.75–0.91]) and all-cause dementia (0.9 [0.84–0.95]). MASLD was associated with an increased risk of vascular dementia (1.24 [1.1–1.39]) but not Alzheimer’s disease (1.0 [0.91–1.09]). The effect of MAFLD on vascular dementia was consistent regardless of MASLD presence, whereas associations with Alzheimer’s disease were only present in those without MASLD (0.78 [0.67–0.91]).

**Conclusions:**

MAFLD and MASLD are associated with an increased risk of vascular dementia, with subtype-specific variations observed in dementia risks. Further research is needed to refine MAFLD and SLD subtyping and explore the underlying mechanisms contributing to dementia risk.

**Supplementary Information:**

The online version contains supplementary material available at 10.1186/s13195-024-01498-5.

## Background

Liver disease accounts for over 2 million global deaths per year, representing approximately 3.5–4% of total worldwide mortality, as estimated in 2015 [1, 2]. Among liver diseases, nonalcoholic fatty liver disease (NAFLD) is a prominent contributor [2, 3]. The term “nonalcoholic” is widely used, but it fails to accurately reflect the disease’s true origins, as there is considerable overlap between NAFLD and alcohol-related liver disease (ALD).

In 2020, a new nomenclature, metabolic dysfunction-associated fatty liver disease (MAFLD), was introduced as an alternative to NAFLD [4]. This updated terminology aims to shift the focus towards recognizing the primary factors driving NAFLD, rather than merely excluding other potential causes [4]. However, MAFLD has not gained universal acceptance, as concerns persist about mixing etiologies and overlooking a significant proportion of NAFLD patients with lean and normal body mass index (BMI) [5]. In 2023, a Delphi consensus statement introduced metabolic dysfunction-associated steatotic liver disease (MASLD) as a new term [6]. Unlike MAFLD, MASLD considers varying levels of alcohol consumption and avoids clinically challenging criteria and biological measurements, such as insulin resistance, which can be difficult to assess in routine clinical practice.

Dementia ranks as the fifth leading cause of death worldwide, with its prevalence on the rise [7]. Despite extensive efforts, the mechanisms underlying dementia remain largely unknown, and effective treatments are lacking. Importantly, there are shared risk factors between dementia and liver diseases. Emerging evidence suggests that the pathological pathways triggered by NAFLD, including insulin resistance, neuroinflammation, hyperammonemia, gut dysbiosis, and cerebrovascular dysfunction, may contribute to dementia development [8].

Some studies have demonstrated an association between NAFLD and reduced brain volume in healthy adults [9, 10], poorer cognitive function across multiple domains [11] and accelerated aging in patients with advanced fibrosis caused by NAFLD [12]. However, a post-hoc analysis of two large cardiovascular trials did not find a significant association between chronic liver disease and brain imaging markers [13]. Several observational studies investigating the link between NAFLD and dementia have produced conflicting results. While a Swedish [14] and a South Korean cohort study [15] suggested a modestly increased risk of vascular dementia in NAFLD individuals, others did not report an association [16–18], or even reported an inverse one [16, 19]. Most of these association studies have limitations, including cross-sectional designs, insufficient adjustments for covariates, small-to-moderate sample sizes, and limited follow-up durations. Moreover, to our knowledge, no prior study has examined or compared the association between the two newly proposed nomenclatures, MAFLD and MASLD, and the risk of dementia.

This study aims to address these gaps by conducting a prospective investigation in a large population-based UK cohort. The objective is to thoroughly explore the independent longitudinal association of MAFLD and/or MASLD with all-cause and cause-specific dementia. Additionally, the study aims to investigate dementia outcomes based on subtypes defined by the MAFLD criteria, as well as subtypes of steatotic liver diseases (SLD) based on the Delphi consensus.

## Methods

### Study designs and participants

The UK Biobank is a large prospective cohort comprising over 500,000 participants aged 38 to 72 years at recruitment between 2006 and 2010 at one of the 22 assessment centers across England, Scotland, and Wales [20]. The study received ethical approval from the North West Multicenter Research Ethics Committee, and all participants provided written informed consent. Participants with an available fatty liver index (FLI) at enrollment were included, and those with pre-existing dementia were excluded. The study was reported according to the STROBE guidelines (Supplementary materials).

### Exposures

Hepatic steatosis was assessed using the FLI, as specific imaging or histological data related to fatty liver were not available in the UK Biobank. The FLI is based on BMI, waist circumference, triglycerides, and gamma-glutamyltransferase (GGT) and has demonstrated reliability as an alternative to imaging techniques such as ultrasonography and transient elastography, showing a good diagnostic performance with an area under the receiver operator curve (AUROC) of 0.85 [21]. An FLI ≥ 60 indicated hepatic steatosis [22].

MAFLD diagnosis relied on hepatic steatosis evidence meeting any of three criteria: overweight/obesity, type 2 diabetes, or at least two metabolic abnormalities. MAFLD had three subtypes: diabetes subtype, overweight/obesity subtype, and lean metabolic disorder subtype [23]. MASLD was defined as the presence of fatty liver along with at least one of the five specified criteria, excluding secondary liver steatosis causes [6]. Subtypes of SLD included MASLD, MetALD (MASLD with higher alcohol intake or other combination etiology), cryptogenic SLD, and SLD with specific etiologies.

### Outcomes

We gathered disease diagnosis information and diagnosis dates from hospital inpatient records and death registry records. Our primary outcomes were incident all-cause dementia, Alzheimer’s disease and vascular dementia. Due to the limited number of incident cases available for obtaining reliable associations, the results for frontotemporal dementia were analyzed but only presented in the supplementary materials. We identified these diagnoses using International Classification of Disease-10 (ICD-10) and ICD-9 codes, with the diagnosis date determined by the earliest date of either primary or secondary diagnosis. The ICD codes for outcomes were shown in supplementary Table 1. The UK Biobank Outcome Adjudication Group conducted outcome adjudication for incident dementia.

### Covariates

In our full-model analyses, we considered the following covariates: age, sex, race, education, smoking and drinking status, Townsend Deprivation Index (TDI), annual household income, physical activity, cardiovascular disease (CVD), and APOE ε4 status. Alcohol intake was categorized as daily or almost daily, 3–4 times per week, 1–2 times per week, occasionally, or never. The TDI is a composite measure reflecting socioeconomic status and categorized into quartiles, with higher scores indicating lower socioeconomic status. Self-reported physical activity was categorized as high, moderate, or low, based on the validated International Physical Activity Questionnaire (IPAQ). CVD was defined as a composite of ischemic heart disease, stroke, and heart failure [14]. *APOE ε4* genotype was defined by two SNPs, rs429358 and rs7412, and categorized as noncarriers (−/−), heterozygotes (+/−), and homozygotes (+/+).

### Statistical analysis

The participants’ baseline characteristics were presented as means ± standard deviation (SD) for continuous variables and as numbers (percentages [%]) for categorical variables. We used Cox proportional hazards regression models to assess the associations of MAFLD or MASLD with the time to incident dementia. In these models, reference groups included participants without MAFLD, without MASLD, without both MAFLD and MASLD, or without hepatic steatosis, as specified. Missing data were coded separately for categorical variables. We examined the proportional hazards assumption by testing Schoenfeld residuals. Follow-up time was calculated from attendance date until the first dementia diagnosis, death, or the censoring date (October 31, 2022), whichever occurred first.

In addition to univariable analysis, we conducted adjusted analyses. Model 1 adjusted for age, sex, race or ethnicity, education, TDI, annual household income, smoking status, alcohol intake, and physical activity. Model 2 further adjusted for CVD and *APOE* status, with covariate selection based on previous literature, clinical relevance, and data availability. Notably, certain covariates such as waist circumference, BMI, diabetes, hypertension, and dyslipidemia were not adjusted for, as they were already incorporated into the definitions of MAFLD and MASLD to avoid overadjustment [24, 25].

We examined the potential interactions between each covariate and MAFLD or MASLD on risk of dementia by including one interaction term at a time in the multivariate models. We performed post-hoc subgroup analyses based on age categories (< 65 years and ≥ 65 years). We also analyzed the association of different MAFLD and SLD subtypes with dementia.

To ensure robustness, we conducted sensitivity analyses. Firstly, we excluded participants who experienced dementia events within the first 2 and 5 years of follow-up to address potential reverse causality. Secondly, to address the potential confounding effect of mixed dementia (involving both Alzheimer’s and vascular dementia), we excluded participants who were diagnosed with vascular dementia before or concurrently with Alzheimer’s dementia during the follow-up period when using Alzheimer’s dementia as the outcome, or vice versa. Lastly, to account for the potential effect modification of death before dementia events, we conducted competing risk analyses using the Fine-Gray proportional subhazards model, treating death from other causes as a competing event [26].

Statistical analyses were performed using SAS software, version 9.4 (SAS Institute Inc, Cary, NC). A two-tailed p-value less than 0.05 was considered statistically significant.

## Results

### Baseline characteristics

**Fig. 1:**
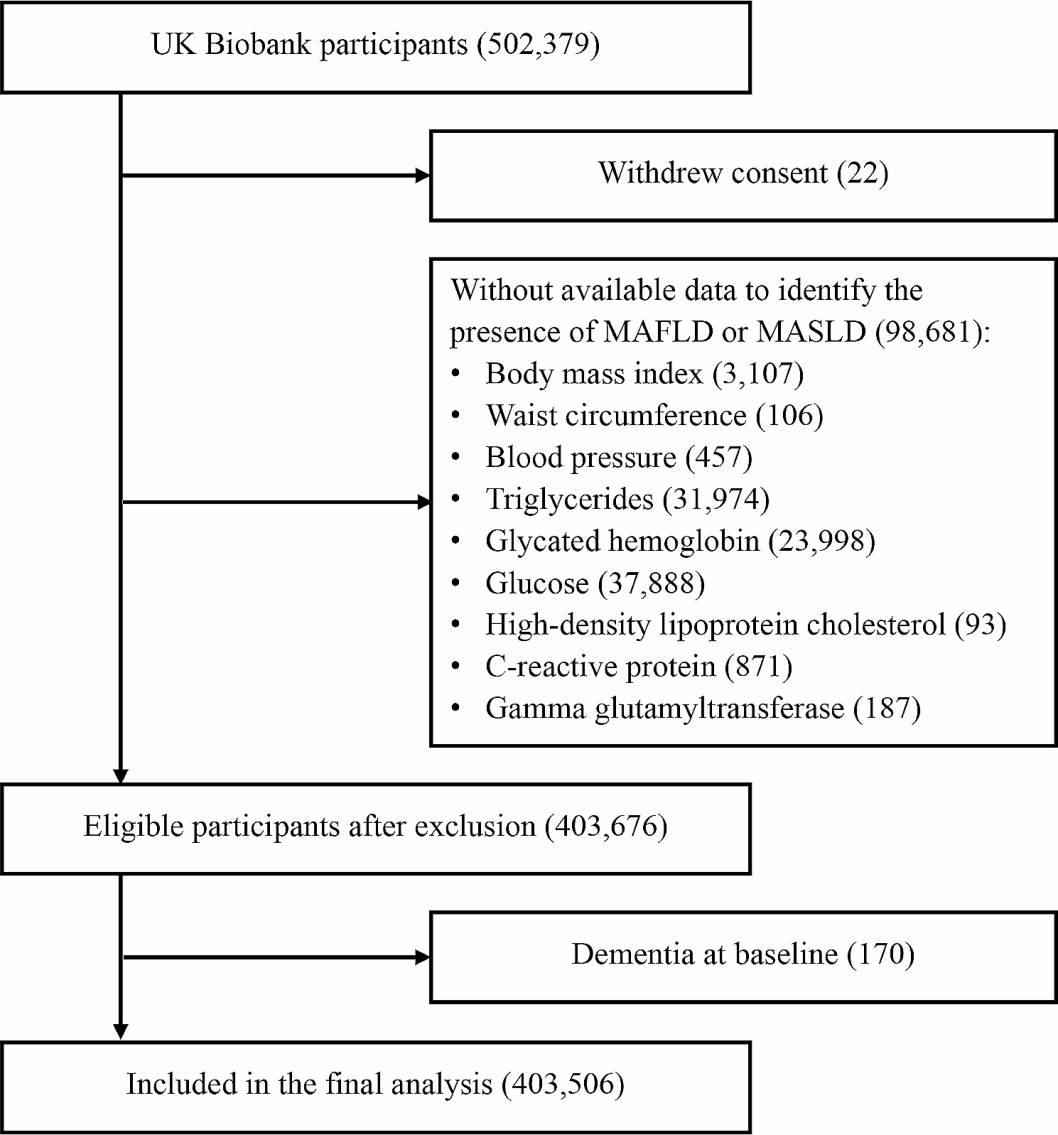
Study population flow chart

A total of 403,506 participants were included in the analyses (Fig. 1). At baseline, the participants had a mean age of 56.6 ± 8.1 years, with 53.8% being females. The baseline characteristics of participants are presented in Table 1. Among the entire cohort, 155,068 (38.4%) participants had MAFLD and 111,938 (27.7%) had MASLD. Among the 155,520 individuals with FLD, 99.7% could be classified as MAFLD, while 72.0% as MASLD; 71.8% were classified as MAFLD + MASLD+, 27.9% MAFLD + MASLD–, and only 0.15% MAFLD–MASLD+.

**Table 1:** Baseline characteristics of study participants according to MAFLD and MASLD status

	Overall (*N* = 403,506)	MAFLD		MASLD	
		No (*N* = 248,438)	Yes (*N* = 155,068)	No (*N* = 291,568)	Yes (*N* = 111,938)
**Age, years**	56.6 ± 8.09	56.1 ± 8.21	57.3 ± 7.83	56.2 ± 8.14	57.4 ± 7.89
**Sex**					
Female	217,162 (53.8)	161,366 (65.0)	55,796 (36.0)	172,060 (59)	45,102 (40.3)
Male	186,344 (46.2)	87,072 (35.1)	99,272 (64.0)	119,508 (41)	66,836 (59.7)
**BMI, kg/m²**					
< 18.5	2,092 (0.52)	2,090 (0.84)	2 (0.00)	2,092 (0.72)	0 (0.00)
≥ 18.5 to < 25.0	130,911 (32.4)	127,776 (51.4)	3,135 (2.02)	128,819 (44.2)	2,092 (1.87)
≥ 25.0 to < 30.0	172,121 (42.7)	108,397 (43.6)	63,724 (41.1)	128,915 (44.2)	43,206 (38.6)
≥ 30.0	98,382 (24.4)	10,175 (4.10)	88,207 (56.9)	31,742 (10.9)	66,640 (59.5)
**Ethnicity**					
White	381,887 (95.1)	235,215 (95.1)	146,672 (95.1)	277,464 (95.6)	104,423 (93.8)
Asian	8,765 (2.18)	5,498 (2.22)	3,267 (2.12)	5,756 (1.98)	3,009 (2.70)
Black	5,338 (1.33)	3,118 (1.26)	2,220 (1.44)	3,323 (1.14)	2,015 (1.81)
Mixed	2,270 (0.57)	1,487 (0.60)	783 (0.51)	1,640 (0.56)	630 (0.57)
Other	3,404 (0.85)	2,066 (0.84)	1,338 (0.87)	2,204 (0.76)	1,200 (1.08)
**Education**					
College or University degree	130,103 (32.3)	89,139 (35.9)	40,964 (26.5)	101,883 (35.0)	28,220 (25.3)
Other	273,018 (67.7)	159,088 (64.1)	113,930 (73.6)	189,469 (65.0)	83,549 (74.8)
**Townsend deprivation index quartile**					
Q1 (least deprived)	100,813 (25.0)	65,413 (26.4)	35,400 (22.9)	75,824 (26.0)	24,989 (22.4)
Q2	100,697 (25.0)	63,767 (25.7)	36,930 (23.9)	74,233 (25.5)	26,464 (23.7)
Q3	100,752 (25.0)	61,983 (25.0)	38,769 (25.0)	72,915 (25.0)	27,837 (24.9)
Q4 (most deprived)	100,752 (25.0)	56,979 (23.0)	43,773 (28.3)	68,254 (23.4)	32,498 (29.1)
**Income levels**					
Level 1 (<£18,000)	78,141 (22.7)	43,476 (20.5)	34,665 (26.1)	51,102 (20.4)	27,039 (28.7)
Level 2 (£8000–30,999)	87,592 (25.4)	53,261 (25.1)	34,331 (25.8)	62,489 (24.9)	25,103 (26.6)
Level 3 (£31,000–52,000)	90,163 (26.1)	56,535 (26.7)	33,628 (25.3)	67,022 (26.7)	23,141 (24.6)
Level 4 (>£52,000)	89,120 (25.8)	58,877 (27.8)	30,243 (22.8)	70,145 (28.0)	18,975 (20.1)
**Smoking status**					
Never	219,250 (54.6)	145,084 (58.7)	74,166 (48.1)	160,347 (55.2)	58,903 (53)
Past	139,726 (34.8)	77,596 (31.4)	62,130 (40.3)	98,712 (34.0)	41,014 (36.9)
Current	42,538 (10.6)	24,697 (10.0)	17,841 (11.6)	31,287 (10.8)	11,251 (10.1)
**Alcohol intake**					
Daily or almost daily	82,300 (20.4)	50,520 (20.4)	31,780 (20.5)	74,798 (25.7)	7,502 (6.70)
3–4 times a week	93,424 (23.2)	59,523 (24.0)	33,901 (21.9)	73,960 (25.4)	19,464 (17.4)
1–2 times a week	104,287 (25.9)	64,870 (26.2)	39,417 (25.5)	68,406 (23.5)	35,881 (32.2)
Occasionally	90,718 (22.5)	54,400 (21.9)	36,318 (23.5)	54,832 (18.8)	35,886 (32.2)
Never	31,946 (7.93)	18,659 (7.52)	13,287 (8.59)	19,076 (6.55)	12,870 (11.5)
**Physical activity**					
Low	61,376 (18.8)	31,259 (15.4)	30,117 (24.4)	39,435 (16.5)	21,941 (25.1)
Moderate	132,994 (40.8)	82,721 (40.8)	50,273 (40.7)	97,488 (40.8)	35,506 (40.6)
High	131,760 (40.4)	88,611 (43.7)	43,149 (34.9)	101,850 (42.7)	29,910 (34.2)
**Diabetes**					
No	378,471 (93.8)	241,748 (97.3)	136,723 (88.2)	281,176 (96.4)	97,295 (86.9)
Yes	25,035 (6.20)	6,690 (2.69)	18,345 (11.8)	10,392 (3.56)	14,643 (13.1)
**Hypertension**					
No	284,757 (70.6)	196,570 (79.1)	88,187 (56.9)	220,624 (75.7)	64,133 (57.3)
Yes	118,749 (29.4)	51,868 (20.9)	66,881 (43.1)	70,944 (24.3)	47,805 (42.7)
**Cardiovascular disease**					
No	375,559 (93.1)	236,547 (95.2)	139,012 (89.7)	275,607 (94.5)	99,952 (89.3)
Yes	27,947 (6.93)	11,891 (4.79)	16,056 (10.4)	15,961 (5.47)	11,986 (10.7)
***APOE ε4*** **carrier**					
−/−	304,591 (75.9)	186,813 (75.6)	117,778 (76.4)	219,705 (75.8)	84,886 (76.3)
+/−	88,694 (22.1)	55,225 (22.4)	33,469 (21.7)	64,450 (22.2)	24,244 (21.8)
+/+	8,051 (2.01)	5,079 (2.06)	2,972 (1.93)	5,891 (2.03)	2,160 (1.94)
Incidence of all-cause dementia	5,732 (1.42)	3,202 (1.29)	2,530 (1.63)	3,808 (1.31)	1,924 (1.72)

Age is presented as mean ± standard deviation. All other data are n or n (%)

BMI, body mass index; MAFLD, metabolic dysfunction associated fatty liver disease; MASLD, metabolic dysfunction associated steatotic liver disease

Compared to participants without MASLD, those with MASLD tended to be older, more likely to be male, physically inactive, and non-White. They also had higher BMI, lower education levels, lower household income, and lower alcohol consumption. The prevalence of hypertension, diabetes, and CVD was higher in participants with MASLD compared to those without MASLD. Most characteristics between the MAFLD + and MASLD + groups were similar, with the exception of a higher percentage of men and greater alcohol consumption in the MAFLD group (Table 1).

### MAFLD, its subtypes and dementia

Over a median follow-up of 13.7 years (interquartile range 12.9–14.4), there were 5,732 new dementia events recorded, including 2,355 cases of Alzheimer’s disease and 1,274 cases of vascular dementia. The proportional hazards assumption was assessed using Schoenfeld residuals, and no violations were found. In the fully adjusted model, MAFLD was associated with a higher risk of vascular dementia (1.32 [1.18–1.48]) but a lower risk of Alzheimer’s disease (0.92 [0.84–1.0]). The risk of all-cause dementia was not statistically different (1.03 [0.98–1.09]) (Table 2).

**Table 2:** Association of MAFLD, its subtypes with all-cause and cause-specific dementia

	No. of participants	No. of cases (%)	Crude model^a^		Multivariable model 1^b^		Multivariable model 2^c^	
			HR (95% CI)	*P* value	HR (95% CI)	*P* value	HR (95% CI)	*P* value
**All-cause dementia**								
MAFLD-	248,438	3,202 (1.29)	Reference	-	Reference	-	Reference	-
MAFLD+	155,068	2,530 (1.63)	1.30 (1.23, 1.37)	< 0.0001	1.08 (1.02, 1.14)	0.0081	1.03 (0.98, 1.09)	0.2556
No SLD	247,986	3,197 (1.29)	Reference	-	Reference	-	Reference	-
Non-MAFLD steatosis	452	5 (1.11)	0.89 (0.37, 2.13)	0.7841	1.03 (0.43, 2.48)	0.9443	1.08 (0.45, 2.60)	0.8593
MAFLD (diabetes)	18,345	672 (3.66)	3.14 (2.89, 3.41)	< 0.0001	2.02 (1.85, 2.20)	< 0.0001	1.80 (1.65, 1.96)	< 0.0001
MAFLD (overweight/obesity)	133,927	1,805 (1.35)	1.06 (1.00, 1.13)	0.0411	0.92 (0.87, 0.98)	0.0060	0.90 (0.84, 0.95)	0.0003
MAFLD (lean metabolic disorder)	2,796	53 (1.90)	1.55 (1.18, 2.03)	0.0016	1.21 (0.92, 1.58)	0.1771	1.21 (0.92, 1.59)	0.1731
**Alzheimer disease**								
MAFLD-	248,438	1,411 (0.57)	Reference	-	Reference	-	Reference	-
MAFLD+	155,068	944 (0.61)	1.10 (1.01, 1.19)	0.0244	0.94 (0.86, 1.02)	0.1548	0.92 (0.84, 1.00)	0.0511
No SLD	247,986	1,409 (0.57)	Reference	-	Reference	-	Reference	-
Non-MAFLD steatosis	452	2 (0.44)	0.80 (0.20, 3.20)	0.7528	1.01 (0.25, 4.06)	0.9846	1.04 (0.26, 4.18)	0.9528
MAFLD (diabetes)	18,345	225 (1.23)	2.38 (2.06, 2.74)	< 0.0001	1.55 (1.35, 1.79)	< 0.0001	1.46 (1.26, 1.69)	< 0.0001
MAFLD (overweight/obesity)	133,927	703 (0.52)	0.94 (0.86, 1.03)	0.1676	0.84 (0.76, 0.92)	0.0002	0.83 (0.75, 0.91)	< 0.0001
MAFLD (lean metabolic disorder)	2,796	16 (0.57)	1.06 (0.65, 1.73)	0.8186	0.87 (0.53, 1.42)	0.5701	0.87 (0.53, 1.42)	0.5734
**Vascular dementia**								
MAFLD-	248,438	602 (0.24)	Reference	-	Reference	-	Reference	-
MAFLD+	155,068	672 (0.43)	1.83 (1.64, 2.05)	< 0.0001	1.43 (1.28, 1.60)	< 0.0001	1.32 (1.18, 1.48)	< 0.0001
No SLD	247,986	601 (0.24)	Reference	-	Reference	-	Reference	-
Non-MAFLD steatosis	452	1 (0.22)	0.94 (0.13, 6.66)	0.9483	1.02 (0.14, 7.26)	0.9845	1.13 (0.16, 8.05)	0.9022
MAFLD (diabetes)	18,345	248 (1.35)	6.12 (5.28, 7.10)	< 0.0001	3.60 (3.09, 4.19)	< 0.0001	2.95 (2.53, 3.45)	< 0.0001
MAFLD(overweight/obesity)	133,927	406 (0.3)	1.27 (1.12, 1.44)	0.0002	1.05 (0.92, 1.19)	0.4715	1.00 (0.88, 1.14)	0.9759
MAFLD (lean metabolic disorder)	2,796	18 (0.64)	2.79 (1.75, 4.46)	< 0.0001	2.00 (1.25, 3.20)	0.0039	2.01 (1.25, 3.22)	0.0037

HR, hazard ratio; MAFLD, metabolic dysfunction associated fatty liver disease; SLD, steatotic liver diseases

^a^Analysis by Cox proportional hazards model

^b^Adjusted for age, sex, ethnicity, education, Townsend deprivation index, income levels, smoking status, alcohol intake and physical activity

^c^Additionally adjusted for *APOE ε4* genotypes and cardiovascular diseases

18,345 individuals were classified as having the MAFLD diabetes subtype, 133,927 as the overweight/obesity subtype, and 2,796 as the lean metabolic disorder subtype. In the full adjustment model, individuals with the MAFLD diabetes subtype had a higher risk of all-cause dementia (1.8 [1.65–1.96]), Alzheimer’s disease (1.46 [1.26–1.69]), and vascular dementia (2.95 [2.52–3.45]) compared to those without hepatic steatosis. Conversely, the MAFLD overweight/obese subtype was associated with a lower risk of all-cause dementia (0.9 [0.84–0.95]), primarily driven by Alzheimer’s disease (0.83 [0.75–0.91]); the risk of vascular dementia did not differ (1.0 [0.88–1.13]). In the MAFLD lean metabolic disorder subtype, there was a higher risk of vascular dementia (2.01 [1.25–3.22]), while the risks of all-cause dementia (1.21 [0.92–1.59]) and Alzheimer’s disease (0.87 [0.53–1.42]) were not significantly different, although the number of dementia events was limited (Table 2).

### MASLD, SLD subtypes and dementia

In the fully adjusted model, MASLD was associated with a higher risk of vascular dementia (1.24 [1.1–1.39]), but the risk of Alzheimer’s disease (1.0 [0.91–1.09]) and all-cause dementia (1.05 [0.99–1.11]) did not differ significantly (Table 3).

**Table 3:** Association of MASLD, SLD subtypes with all-cause and cause-specific dementia

	No. of participants	No. of cases (%)	Crude model^a^		Multivariable model 1^b^		Multivariable model 2^c^	
			HR (95% CI)	*P* value	HR (95% CI)	*P* value	HR (95% CI)	*P* value
**All-cause dementia**								
MASLD–	291,568	3,808 (1.31)	Reference	-	Reference	-	Reference	-
MASLD+	111,938	1,924 (1.72)	1.34 (1.27, 1.42)	< 0.0001	1.09 (1.03, 1.15)	0.0046	1.05 (0.99, 1.11)	0.1173
No SLD	247,986	3,197 (1.29)	Reference	-	Reference	-	Reference	-
MASLD	111,938	1,924 (1.72)	1.37 (1.29, 1.45)	< 0.0001	1.09 (1.03, 1.16)	0.0037	1.05 (0.99, 1.11)	0.1382
MetALD	43,528	610 (1.4)	1.12 (1.03, 1.22)	0.0106	1.03 (0.94, 1.13)	0.5291	0.99 (0.91, 1.09)	0.8581
Cryptogenic SLD	30	0 (0.00)	-	-	-	-	-	-
Other specific etiology SLD	24	1 (4.17)	4.32 (0.61, 30.7)	0.1437	6.86 (0.97, 48.8)	0.0542	5.17 (0.73, 36.8)	0.1005
**Alzheimer’s disease**								
MASLD–	291,568	1,609 (0.55)	Reference	-	Reference	-	Reference	-
MASLD+	111,938	746 (0.67)	1.23 (1.13, 1.34)	< 0.0001	1.02 (0.93, 1.11)	0.7405	1.00 (0.91, 1.09)	0.9122
No SLD	247,986	1,409 (0.57)	Reference	-	Reference	-	Reference	-
MASLD	111,938	746 (0.67)	1.20 (1.10, 1.31)	< 0.0001	0.99 (0.9, 1.08)	0.7709	0.96 (0.88, 1.06)	0.4151
MetALD	43,528	200 (0.46)	0.83 (0.72, 0.97)	0.0152	0.80 (0.69, 0.93)	0.0044	0.79 (0.67, 0.92)	0.0019
Cryptogenic SLD	30	0 (0.00)	-	-	-	-	-	-
Other specific etiology SLD	24	0 (0.00)	-	-	-	-	-	-
**Vascular dementia**								
MASLD–	291,568	772 (0.26)	Reference	-	Reference	-	Reference	-
MASLD+	111,938	502 (0.45)	1.73 (1.54, 1.93)	< 0.0001	1.34 (1.19, 1.50)	< 0.0001	1.24 (1.10, 1.39)	0.0003
No SLD	247,986	601 (0.24)	Reference	-	Reference	-	Reference	-
MASLD	111,938	502 (0.45)	1.89 (1.68, 2.13)	< 0.0001	1.43 (1.26, 1.62)	< 0.0001	1.32 (1.16, 1.49)	< 0.0001
MetALD	43,528	170 (0.39)	1.66 (1.40, 1.97)	< 0.0001	1.42 (1.19, 1.70)	0.0001	1.33 (1.12, 1.59)	0.0015
Cryptogenic SLD	30	0 (0.00)	-	-	-	-	-	-
Other specific etiology SLD	24	1 (4.17)	22.8 (3.21, 162.2)	0.0018	36.1 (5.06, 257.1)	0.0003	21.5 (3.01, 153.5)	0.0022

HR, hazard ratio; MASLD, metabolic dysfunction-associated steatotic liver disease; MetALD, MASLD with greater alcohol consumption; SLD, steatotic liver diseases

^a^Analysis by Cox proportional hazards model

^b^Adjusted for age, sex, ethnicity, education, Townsend deprivation index, income levels, smoking status, alcohol intake and IPAQ activity group

^c^Additionally adjusted for *APOE ε4* genotypes and cardiovascular diseases

111,938 individuals had MASLD, 43,528 had MetALD, 30 had cryptogenic SLD, and 24 had other specific etiology SLD. After full adjustment, individuals with MASLD had a higher risk of vascular dementia (1.32 [1.16–1.49]) compared to those without hepatic steatosis, but the risk of other types of dementia was not statistically different. MetSLD was associated with a lower risk of Alzheimer’s disease (0.79 [0.67–0.92]), but a higher risk of vascular dementia (1.33 [1.12–1.59]), while the risk of all-cause dementia was similar. The number of participants with cryptogenic SLD and other specific etiology SLD was small (Table 3).

### MAFLD, MASLD combinations and dementia

After full adjustment, compared to MAFLD–MASLD–, MAFLD + MASLD– was associated with a higher risk of vascular dementia (1.33 [1.12–1.59]), but a decreased risk of Alzheimer’s disease (0.78 [0.67–0.91]). Similarly, MAFLD + MASLD + was also associated with a higher risk of vascular dementia (1.31 [1.16–1.49]), but the risk of Alzheimer’s disease (0.96 [0.88–1.06]) was similar. The risk of all-cause dementia did not show statistically significant differences between MAFLD + MASLD– and MAFLD–MASLD–, as well as between MAFLD + MASLD + and MAFLD–MASLD– (Table 4).

**Table 4:** Risk of all-cause and cause-specific dementia according to presence and combination of MAFLD and/or MASLD.

	No. of participants	No. of cases (%)	Crude model^a^		Multivariable model 1^b^		Multivariable model 2^c^	
			HR (95% CI)	*P* value	HR (95% CI)	*P* value	HR (95% CI)	*P* value
**All-cause dementia**								
MAFLD–/MASLD–	248,213	3,202 (1.29)	Reference	-	Reference	-	Reference	-
MAFLD+/MASLD–	43,355	606 (1.40)	1.12 (1.02, 1.22)	0.0135	1.02 (0.94, 1.12)	0.6088	0.99 (0.90, 1.08)	0.7665
MAFLD–/MASLD+	225	0 (0.00)	-	-	-	-	-	-
MAFLD+/MASLD+	111,713	1,924 (1.72)	1.37 (1.29, 1.45)	< 0.0001	1.09 (1.03, 1.16)	0.0035	1.05 (0.99, 1.11)	0.1353
**Alzheimer’s disease**								
MAFLD–/MASLD–	248,213	1,411 (0.57)	Reference	-	Reference	-	Reference	-
MAFLD+/MASLD–	43,355	198 (0.46)	0.83 (0.71, 0.96)	0.0123	0.79 (0.68, 0.93)	0.0032	0.78 (0.67, 0.91)	0.0014
MAFLD–/MASLD+	225	0 (0.00)	-	-	-	-	-	-
MAFLD+/MASLD+	111,713	746 (0.67)	1.20 (1.10, 1.32)	< 0.0001	0.99 (0.90, 1.08)	0.7796	0.96 (0.88, 1.06)	0.4204
**Vascular dementia**								
MAFLD–/MASLD–	248,213	602 (0.24)	Reference	-	Reference	-	Reference	-
MAFLD+/MASLD–	43,355	170 (0.39)	1.66 (1.40, 1.97)	< 0.0001	1.42 (1.19, 1.70)	0.0001	1.33 (1.12, 1.59)	0.0015
MAFLD–/MASLD+	225	0 (0.00)	-	-	-	-	-	-
MAFLD+/MASLD+	111,713	502 (0.45)	1.90 (1.68, 2.13)	< 0.0001	1.43 (1.26, 1.62)	< 0.0001	1.31 (1.16, 1.49)	< 0.0001

HR, hazard ratio; MAFLD, metabolic dysfunction associated fatty liver disease; MASLD, metabolic dysfunction–associated steatotic liver disease

^a^Analysis by Cox proportional hazards model

^b^Adjusted for age, sex, ethnicity, education, Townsend deprivation index, income levels, smoking status, alcohol intake and IPAQ activity group

^c^Additionally adjusted for *APOE ε4* genotypes and cardiovascular diseases

### Subgroup and sensitivity analyses

No significant association was detected between MAFLD, MASLD, their combinations or subgroups with frontotemporal dementia (supplementary Table 2). The overall results remained consistent when the analyses were stratified by various factors. A significant interaction was found between MAFLD and age in relation to the risk of all-cause and vascular dementia, and between MASLD and age regarding vascular dementia. Therefore, we conducted post-hoc subgroup analyses based on age categories (< 65 years and ≥ 65 years). Generally, we found associations with dementia being more prominent in groups with younger age (supplementary Tables 3–5). Consistent results were observed when considering only dementia events that occurred at least 2 or 5 years after baseline (supplementary Tables 6–11). Additionally, taking into account the competing risk of death from other causes (supplementary Tables 12–14) or excluding participants with mixed dementia (supplementary Tables 15–17) also showed comparable findings.

## Discussions

In this ∼13-year follow-up study of 403,506 participants from UK Biobank, MAFLD was associated with a higher risk of vascular dementia but a lower risk of Alzheimer’s disease. The increased risk of vascular dementia was primarily driven by the presence of diabetes and lean metabolic disorder subtypes within the MAFLD group. Conversely, the lower risk of Alzheimer’s disease was mainly attributed to the protective effects seen in the overweight/obesity subtype of MAFLD. MASLD was also associated with a higher risk of vascular dementia but did not affect the risk of Alzheimer’s disease. The impact of MAFLD on vascular dementia was consistent regardless of the presence of MASLD, but the effects on Alzheimer’s disease were only evident in individuals without MASLD. Neither MAFLD, MASLD nor the combinations of MAFLD and MASLD had a significant impact on the risk of all-cause dementia, except that MAFLD diabetes subtype was associated with a higher risk of all-cause dementia.

The prevalence of MAFLD in the UK Biobank population was 38.4%, with MASLD prevalence at 27.7%. These rates were higher than those reported in the NHANES III (20.4% and 14.9%, respectively) [24], but similar to rates in a national cohort in Korea (37.3% for MAFLD) [25]. It is noteworthy that almost all MASLD subjects (99.8%) fell within the MAFLD diagnosis, wheras only 72.0% of MAFLD cases fell within MASLD. MAFLD+/MASLD- individuals have hepatic steatosis with concurrent liver disease or exhibit only two components of metabolic syndrome (insulin resistance assessed by HOMA and inflammation assessed by CRP levels) [23]. This relatively high percentage underscores the diverse etiologies underlying MAFLD. On the other hand, MAFLD–/MASLD + individuals have hepatic steatosis with only one component of specific cardiometabolic criteria (prediabetes, hypertension, increased waist circumference, increased triglycerides, or decreased HDL cholesterol) [6]. The notebly lower percentage of this group suggests that individuals with a singular cardiometabolic abnormality often exhibit coexistence with other cardiometabolic abnormalities within the UK population.

Our study represents the first longitudinally investigation into the association of MAFLD and MASLD with all-cause and cause-specific dementia. We found a positive link between MAFLD and vascular dementia, primarily driven by diabetes and the lean metabolic disorder subtypes. Similarly, MASLD and MetALD were also associated with vascular dementia, indicating that hepatic steatosis, diabetes, lean metabolic syndrome, and excessive alcohol consumption collectively contribute to the risk of vascular dementia. However, the overweight/obese MAFLD subtype did not follow this pattern. A study utilizing NHANES III databases similarly found that the overweight/obese MAFLD subtype was not associated with increased risk of all-cause mortality, unlike the other two subtypes [27]. These varying prognostic effects of the MAFLD subtypes on mortality [27], myocardial infarction [28], and dementia highlight the heterogeneity of MAFLD definitions and emphasize the importance for further subclassification to guide tailored therapeutic interventions.

The inverse relationship between MAFLD and Alzheimer’s disease was predominantly observed in the overweight/obesity subtype. Our sensitivity analyses, focusing on dementia events occurring after 5 years of follow-up, provided consistent results and minimized the likelihood of reverse causation between obesity and dementia. The relatioinship between BMI and dementia events is complex and has produced conflicting findings in prior research. Some studies suggest that being overweight in mid-life increases the risk of dementia later in life. However, in later life, being overweight may be associated with a reduced dementia risk [29], exemplifying the obesity paradox [30]. Nevertheless, other studies present divergent results. For example, a large cohort study involving 1,958,191 UK participants and 45,507 dementia events found that dementia incidence decreased with increasing BMI categories [31], even after excluding events within 15 years [31]. Among participants aged 60 years and older, a higher BMI was associated with a reduced risk of Alzheimer’s disease among those with the same genetic risk [32]. Furthermore, declining BMI has been associated with an increased risk of incident Alzheimer’s disease [33], while weight gain is associated with reduced dementia-related mortality [34].

MetALD, a subtype of SLD within the new consensus, was also associated with a reduced risk of Alzheimer’s disease. This suggests that MetALD, which is largely included in the MAFLD definition but not separately classified, contributes significantly to the risk reduction of Alzheimer’s disease associated with MAFLD, alongside the overweight/obesity subtype. While MASLD was not associated with a decreased risk of Alzheimer’s disease, the effect of observed with MetALD may stem from alcohol consumption or the interaction between alcohol and metabolic dysfunction. The lower risk of Alzheimer’s disease in MAFLD + MASLD– individuals compared to MAFLD–MASLD– individuals supports this speculation, as the MAFLD + MASLD– group largely represents individuals with concomitant alcohol liver disease and metabolic abnormalities.

The relationship between alcohol consumption and dementia is complex and inconclusive. Studies have reported a 22% reduction in the risk of Alzheimer’s disease among mild-to-moderate alcohol consumers compared to non-consumers [35, 36], which aligns with our findings. This protective effect may be attributed to mechanisms such as prosurvival pathways promotion and reduction in neuroinflammation [37]. Conversely, sustained heavy drinking is associated with a significantly increased risk of Alzheimer’s disease [36].

Research often demonstrates a J-shaped or U-shaped association between alcohol consumption and all-cause dementia risk, but the threshold for dementia risk remains uncertain [36, 38]. Establishing a definitive cause-and-effect association between alcohol and Alzheimer’s disease is challenging due to methodological differences across studies, particularly in how alcohol consumption is measured and control groups are defined.

In our study, we did not investigate the amount or type of alcohol consumed. Given the differing effects of MAFLD + MASLD– and MAFLD + MASLD + on Alzheimer’s disease, it is important to distinguish MAFLD with concomitant liver disease from those without in future studies. Although the newly established Delphi consensus statement has addressed this issue to some extent, there is still room for refinement in predicting dementia risk. A more nuanced approach, similar to the subcategorization used in the MAFLD definition, holds promise for improving the accuracy of dementia risk prediction in clinicalsettings.

This study has several strengths, including its prospective design, large sample size, extensive long-term follow-up, robust adjustment for potential confounding factors including genetic background, and the use of multiple sources to identify incident dementia cases. Dementia outcome has been previously validated, demonstrating a sensitivity of 78% and specificity of 92% for dementia diagnosis recording in general hospitals, using secondary mental health care diagnostic status as the gold-standard [39].

However, it’s important to acknowledge some limitations. Firstly, despite using multiple sources to identify incident dementia cases, there is a possibility that early-stage or milder cases of dementia from primary care were missed. Nevertheless, the overall accuracy of dementia diagnosis showed good agreement with primary care records. Secondly, hepatic steatosis was defined using the FLI rather than liver biopsy or imaging. However, the FLI has demonstrated a strong correlation with ultrasound diagnosis of NAFLD in multiple studies [21, 40]. Thirdly, while we controlled for a wide range of confounders, we acknowledge that potential residual confounding cannot be completely ruled out due to the observational nature of the study. Fourthly, the assessment of MAFLD and MASLD was conducted only at baseline, and there is a lack of data regarding exposure durations and any changes during the follow-up period. Fifthly, it’s essential to note that the findings are observational, and therefore, causality cannot be established. Finally, the majority of participants in the UK Biobank study were of White ethnicity, so generalizing the findings to other ethnic groups should be done with caution.

## Conclusions

In conclusion, MAFLD and MASLD are associated with an increased risk of vascular dementia, with subtype-specific variations observed in dementia risks. The MAFLD diabetes subtype elevates the risk of all-cause dementia, vascular dementia, and Alzheimer’s disease, while the lean metabolic disorder subtype only increases the risk of vascular dementia. On the other hand, the overweight/obesity subtype is associated with a reduced risk of Alzheimer’s disease and all-cause dementia. MASLD does not influence the risk of Alzheimer’s disease, but MetSLD is associated with a lower risk of Alzheimer’s disease. Further research is needed to refine MAFLD and SLD subtyping and explore the underlying mechanisms contributing to dementia risk.

### Electronic supplementary material

Below is the link to the electronic supplementary material.

Supplementary Material 1

## Data Availability

Data are available in a public, open access repository. Data from the UK Biobank (https://www.ukbiobank.ac.uk/) are available to researchers on application.

## Abbreviations

ALD: Alcohol-related liver disease
BMI: Body mass index
CVD: Cardiovascular diseases
FLI: Fatty liver index
HR: Hazard ratio
IPAQ: International physical activity questionnaire
MAFLD: Metabolic dysfunction associated fatty liver disease
MASLD: Metabolic dysfunction associated steatotic liver disease
NAFLD: Nonalcoholic fatty liver disease
SLD: Steatotic liver diseases
TDI: Townsend Deprivation Index
