# Supplementary material for: Association of MAFLD and MASLD with all-cause and cause-specific dementia: a prospective cohort study
Source: Alzheimers Res Ther. 2024 Jun 26;16:136. doi: 10.1186/s13195-024-01498-5 (PMC11201326; doi:10.1186/s13195-024-01498-5)
Supplement: Supplementary file 1 — Supplementary Material 1 [file 13195_2024_1498_MOESM1_ESM.docx]

**SUPPLEMENTARY MATERIALS**

**Association of MAFLD and MASLD with All-Cause and Cause-Specific Dementia: A Prospective Cohort Study**

Contents

[Supplementary Table 1. ICD codes for dementia outcomes. 4](#_Toc152141376)

[Supplementary Table 2. Risk of frontotemporal dementia according to the presence of MAFLD and its subtypes, or MASLD and SLD subtypes, or the presence and combination of MAFLD and/or MASLD. 5](#_Toc152141377)

[Supplementary Table 3. The interaction between the presence of MAFLD and other variables in terms of the risk of all-cause and cause-specific dementia. 7](#_Toc152141378)

[Supplementary Table 4. The interaction between the presence of MASLD and other variables in terms of the risk of all-cause and cause-specific dementia. 8](#_Toc152141379)

[Supplementary Table 5. Subgroup analysis stratified by variables with significant interaction effects. 9](#_Toc152141380)

[Supplementary Table 6. Association of MAFLD and its subtypes with all-cause and cause-specific dementia excluding participants with incident dementia within the first 2 years of follow-up. 10](#_Toc152141381)

[Supplementary Table 7. Association of MASLD and SLD subtypes with all-cause and cause-specific dementia excluding participants with incident dementia within the first 2 years of follow-up. 12](#_Toc152141382)

[Supplementary Table 8. Risk of all-cause and cause-specific dementia according to presence and combination of MAFLD and/or MASLD excluding participants with incident dementia within the first 2 years of follow-up. 14](#_Toc152141383)

[Supplementary Table 9. Association of MAFLD and its subtypes with all-cause and cause-specific dementia excluding participants with incident dementia within the first 5 years of follow-up. 16](#_Toc152141384)

[Supplementary Table 10. Association of MASLD and SLD subtypes with all-cause and cause-specific dementia excluding participants with incident dementia within the first 5 years of follow-up. 19](#_Toc152141385)

[Supplementary Table 11. Risk of all-cause and cause-specific dementia according to presence and combination of MAFLD and/or MASLD excluding participants with incident dementia within the first 5 years of follow-up. 21](#_Toc152141386)

[Supplementary Table 12. Association of MAFLD and its subtypes with all-cause and cause-specific dementia taking account competing risk of death from other causes. 23](#_Toc152141387)

[Supplementary Table 13. Association of MASLD and SLD subtypes with all-cause and cause-specific dementia taking account competing risk of death from other causes. 26](#_Toc152141388)

[Supplementary Table 14. Risk of all-cause and cause-specific dementia according to presence and combination of MAFLD and/or MASLD taking account competing risk of death from other causes. 28](#_Toc152141389)

[Supplementary Table 15. Association of MAFLD and its subtypes with Alzheimer’s (or vascular) dementia excluding participants previously or simultaneously diagnosed with vascular (or Alzheimer’s) dementia during the follow-up period. 30](#_Toc152141390)

[Supplementary Table 16. Association of MASLD and SLD subtypes with Alzheimer's (or vascular) dementia excluding participants previously or simultaneously diagnosed with vascular (or Alzheimer's) dementia during the follow-up period. 32](#_Toc152141391)

[Supplementary Table 17. Risk of Alzheimer's (or vascular) dementia according to presence and combination of MAFLD and/or MASLD excluding participants previously or simultaneously diagnosed with vascular (or Alzheimer's) dementia during the follow-up period. 34](#_Toc152141392)

[STROBE Statement—Checklist of items that should be included in reports of cohort studies 35](#_Toc152141393)

# Supplementary Table 1. ICD codes for dementia outcomes.

| Outcomes | ICD codes |
| --- | --- |
| All-cause dementia | ICD-10: A810, F00, F000, F001, F002, F009, F01, F010, F011, F012, F013, F018, F019, F02, F020, F021, F022, F023, F024, F028, F03, F051, F106, G30, G300, G301, G308, G309, G310, G311, G318, I673  ICD-9: 2901, 2902, 2903, 2904, 2912, 2941, 3310, 3311, 3312, 3315 |
| Alzheimer’s disease | ICD-10: F00, F000, F001, F002, F009, G30, G300, G301, G308, G309  ICD-9: 3310 |
| Vascular dementia | ICD-10: F010, F011, F012, F013, F018, F019, I673  ICD-9: 2904 |
| Frontotemporal dementia | ICD-10: F020, G310  ICD-9: 3311 |

# Supplementary Table 2. Risk of frontotemporal dementia according to the presence of MAFLD and its subtypes, or MASLD and SLD subtypes, or the presence and combination of MAFLD and/or MASLD.

|  | **No. of participants** | **No. of cases (%)** | **Crude model** | | **Multivariable model 1^b^** | | **Multivariable model 2^c^** | |
| --- | --- | --- | --- | --- | --- | --- | --- | --- |
|  |  |  | **HR (95% CI)** | ***P* value^a^** | **HR (95% CI)** | ***P* value^a^** | **HR (95% CI)** | ***P* value^a^** |
| **MAFLD and its subtypes** |  |  |  |  |  |  |  |  |
| MAFLD– | 248,438 | 110 (0.04) | Reference | - | Reference | - | Reference | - |
| MAFLD+ | 155,068 | 70 (0.05) | 1.04 (0.77, 1.41) | 0.7865 | 0.84 (0.62, 1.15) | 0.2868 | 0.84 (0.61, 1.14) | 0.2660 |
| No SLD | 247,986 | 110 (0.04) | Reference | - | Reference | - | Reference | - |
| Non-MAFLD steatosis | 452 | 0 (0.00) | - | - | - | - | - | - |
| MAFLD (diabetes) | 18,345 | 11 (0.06) | 1.46 (0.79, 2.72) | 0.2285 | 0.94 (0.50, 1.77) | 0.8544 | 0.92 (0.49, 1.73) | 0.7942 |
| MAFLD (overweight/obesity) | 133,927 | 58 (0.04) | 0.99 (0.72, 1.36) | 0.9501 | 0.83 (0.60, 1.15) | 0.2666 | 0.83 (0.60, 1.15) | 0.2550 |
| MAFLD (lean metabolic disorder) | 2,796 | 1 (0.04) | 0.84 (0.12, 6.05) | 0.8660 | 0.65 (0.09, 4.65) | 0.6648 | 0.65 (0.09, 4.65) | 0.6647 |
| **MASLD and SLD subtypes** |  |  |  |  |  |  |  |  |
| MASLD– | 291,568 | 129 (0.04) | Reference | - | Reference | - | Reference | - |
| MASLD+ | 111,938 | 51 (0.05) | 1.05 (0.76, 1.45) | 0.7820 | 0.79 (0.57, 1.12) | 0.1828 | 0.79 (0.56, 1.11) | 0.1709 |
| No SLD | 247,986 | 110 (0.04) | Reference | - | Reference | - | Reference | - |
| MASLD | 111,938 | 51 (0.05) | 1.05 (0.75, 1.46) | 0.7827 | 0.79 (0.56, 1.12) | 0.1903 | 0.79 (0.56, 1.11) | 0.1766 |
| MetALD | 43,528 | 19 (0.04) | 1.01 (0.62, 1.65) | 0.9651 | 1.00 (0.59, 1.69) | 0.9993 | 0.99 (0.59, 1.67) | 0.9759 |
| Cryptogenic SLD | 30 | 0 (0.00) | - | - | - | - | - | - |
| Other specific etiology SLD | 24 | 0 (0.00) | - | - | - | - | - | - |
| **The combination of MAFLD and/or MASLD** |  |  |  |  |  |  |  |  |
| MAFLD–/MASLD– | 248,213 | 110 (0.04) | Reference | - | Reference | - | Reference | - |
| MAFLD+/MASLD– | 43,355 | 19 (0.04) | 1.02 (0.62, 1.65) | 0.9497 | 1.01 (0.6, 1.69) | 0.9847 | 1.00 (0.59, 1.68) | 0.9906 |
| MAFLD–/MASLD+ | 225 | 0 (0.00) | - | - | - | - | - | - |
| MAFLD+/MASLD+ | 111,713 | 51 (0.05) | 1.05 (0.75, 1.47) | 0.7695 | 0.80 (0.56, 1.12) | 0.1952 | 0.79 (0.56, 1.12) | 0.1813 |

HR, hazard ratio; MAFLD, metabolic dysfunction associated fatty liver disease; MASLD, metabolic dysfunction-associated steatotic liver disease; MetALD, MASLD with greater alcohol consumption; SLD, steatotic liver diseases.

^a^ Analysis by Cox proportional hazards model.

^b^ Adjusted for age, sex, ethnicity, education, Townsend deprivation index, income levels, smoking status, alcohol intake and IPAQ activity group.

^c^ Additionally adjusted for *APOE ε4* genotypes and cardiovascular diseases.

# Supplementary Table 3. The interaction between the presence of MAFLD and other variables in terms of the risk of all-cause and cause-specific dementia.

| **Interaction term** | **All-cause dementia** | **Alzheimer disease** | **Vascular dementia** | **Frontotemporal dementia** |
| --- | --- | --- | --- | --- |
| MAFLD*Age | 0.0019 | 0.216 | 0.0016 | 0.842 |
| MAFLD*Sex | 0.077 | 0.765 | 0.845 | 0.217 |
| MAFLD*Ethnicity | 0.561 | 0.844 | 0.261 | 0.212 |
| MAFLD*Education | 0.346 | 0.370 | 0.213 | 0.966 |
| MAFLD*TDI quartiles | 0.114 | 0.607 | 0.904 | 0.581 |
| MAFLD*Income levels | 0.327 | 0.622 | 0.318 | 0.567 |
| MAFLD*Smoking status | 0.769 | 0.649 | 0.750 | 0.659 |
| MAFLD*Alcohol intake | 0.920 | 0.709 | 0.222 | 0.711 |
| MAFLD*IPAQ activity group | 0.623 | 0.859 | 0.302 | 0.628 |
| MAFLD**APOE ε4* carrier | 0.794 | 0.296 | 0.797 | 0.063 |
| MAFLD*cardiovascular disease | 0.345 | 0.361 | 0.446 | 0.297 |

Data are presented as p value for interaction.

MAFLD, metabolic dysfunction associated fatty liver disease; TDI, Townsend deprivation index.

# Supplementary Table 4. The interaction between the presence of MASLD and other variables in terms of the risk of all-cause and cause-specific dementia.

| **Interaction term** | **All-cause dementia** | **Alzheimer disease** | **Vascular dementia** | **Frontotemporal dementia** |
| --- | --- | --- | --- | --- |
| MASLD*Age | 0.089 | 0.839 | 0.0069 | 0.874 |
| MASLD*Sex | 0.280 | 0.735 | 0.839 | 0.652 |
| MASLD*Ethnicity | 0.179 | 0.855 | 0.310 | 0.285 |
| MASLD*Education | 0.292 | 0.307 | 0.224 | 0.896 |
| MASLD*TDI quartiles | 0.314 | 0.845 | 0.114 | 0.644 |
| MASLD*Income levels | 0.132 | 0.757 | 0.351 | 0.691 |
| MASLD*Smoking status | 0.574 | 0.767 | 0.966 | 0.884 |
| MASLD*Alcohol intake | 0.746 | 0.921 | 0.236 | 0.162 |
| MASLD*IPAQ activity group | 0.422 | 0.929 | 0.548 | 0.252 |
| MASLD**APOE ε4* carrier | 0.262 | 0.245 | 0.362 | 0.131 |
| MASLD*cardiovascular disease | 0.145 | 0.116 | 0.613 | 0.482 |

Data are presented as p value for interaction.

MASLD, metabolic dysfunction associated steatotic liver disease; TDI, Townsend deprivation index.

# Supplementary Table 5. Subgroup analysis stratified by variables with significant interaction effects.

|  | **No. of participants** | **No. of cases (%)** | **Crude model** | | **Multivariable model 1^b^** | | **Multivariable model 2^c^** | |
| --- | --- | --- | --- | --- | --- | --- | --- | --- |
|  |  |  | **HR (95% CI)** | ***P* value^a^** | **HR (95% CI)** | ***P* value^a^** | **HR (95% CI)** | ***P* value^a^** |
| **MAFLD and all-cause dementia** |  |  |  |  |  |  |  |  |
| <65 years | 326,210 | 2,355 | 1.40 (1.29, 1.52) | <0.0001 | 1.26 (1.15, 1.37) | <0.0001 | 1.16 (1.06, 1.26) | 0.0010 |
| ≥65 years | 77,296 | 3,377 | 1.11 (1.04, 1.19) | 0.0021 | 1.04 (0.97, 1.11) | 0.3052 | 1.00 (0.93, 1.07) | 0.9859 |
| **MAFLD and vascular dementia** |  |  |  |  |  |  |  |  |
| <65 years | 326,210 | 456 | 2.30 (1.91, 2.77) | <0.0001 | 1.86 (1.54, 2.26) | <0.0001 | 1.62 (1.33, 1.97) | <0.0001 |
| ≥65 years | 77,296 | 818 | 1.45 (1.27, 1.66) | <0.0001 | 1.30 (1.12, 1.49) | 0.0004 | 1.21 (1.05, 1.39) | 0.0100 |
| **MASLD and vascular dementia** |  |  |  |  |  |  |  |  |
| <65 years | 326,210 | 456 | 2.08 (1.73, 2.50) | <0.0001 | 1.72 (1.43, 2.09) | <0.0001 | 1.51 (1.25, 1.83) | <0.0001 |
| ≥65 years | 77,296 | 818 | 1.35 (1.17, 1.55) | <0.0001 | 1.20 (1.03, 1.39) | 0.0177 | 1.12 (0.97, 1.30) | 0.1324 |

HR, hazard ratio; MAFLD, metabolic dysfunction associated fatty liver disease; MASLD, metabolic dysfunction-associated steatotic liver disease.

Analysis by Cox proportional hazards model.

^a^ Analysis by Cox proportional hazards model.

^b^ Adjusted for sex, ethnicity, education, Townsend deprivation index, income levels, smoking status, alcohol intake and IPAQ activity group.

^c^ Additionally adjusted for *APOE ε4* genotypes and cardiovascular diseases.

# Supplementary Table 6. Association of MAFLD and its subtypes with all-cause and cause-specific dementia excluding participants with incident dementia within the first 2 years of follow-up.

|  | **No. of participants** | **No. of cases (%)** | **Crude model** | | **Multivariable model 1^b^** | | **Multivariable model 2^c^** | |
| --- | --- | --- | --- | --- | --- | --- | --- | --- |
|  |  |  | **HR (95% CI)** | ***P* value^a^** | **HR (95% CI)** | ***P* value^a^** | **HR (95% CI)** | ***P* value^a^** |
| **All-cause dementia** |  |  |  |  |  |  |  |  |
| MAFLD– | 248,397 | 3,161 (1.27) | Reference | - | Reference | - | Reference | - |
| MAFLD+ | 155,034 | 2,496 (1.61) | 1.30 (1.23, 1.37) | <0.0001 | 1.08 (1.02, 1.14) | 0.0075 | 1.03 (0.98, 1.09) | 0.2333 |
| No SLD | 247,946 | 3,157 (1.27) | Reference | - | Reference | - | Reference | - |
| Non-MAFLD steatosis | 451 | 4 (0.89) | 0.72 (0.27, 1.91) | 0.5059 | 0.84 (0.31, 2.24) | 0.7244 | 0.88 (0.33, 2.35) | 0.7969 |
| MAFLD (diabetes) | 18,335 | 662 (3.61) | 3.14 (2.89, 3.41) | <0.0001 | 2.02 (1.85, 2.20) | <0.0001 | 1.81 (1.66, 1.97) | <0.0001 |
| MAFLD (overweight/obesity) | 133,903 | 1,781 (1.33) | 1.06 (1.00, 1.13) | 0.0445 | 0.92 (0.87, 0.98) | 0.0067 | 0.90 (0.85, 0.95) | 0.0004 |
| MAFLD (lean metabolic disorder) | 2,796 | 53 (1.90) | 1.57 (1.20, 2.06) | 0.0011 | 1.22 (0.93, 1.61) | 0.1466 | 1.23 (0.93, 1.61) | 0.1431 |
| **Alzheimer’s disease** |  |  |  |  |  |  |  |  |
| MAFLD– | 248,397 | 1,404 (0.57) | Reference | - | Reference | - | Reference | - |
| MAFLD+ | 155,034 | 942 (0.61) | 1.10 (1.02, 1.20) | 0.0205 | 0.94 (0.87, 1.03) | 0.176 | 0.92 (0.85, 1.00) | 0.0599 |
| No SLD | 247,946 | 1,402 (0.57) | Reference | - | Reference | - | Reference | - |
| Non-MAFLD steatosis | 451 | 2 (0.44) | 0.81 (0.2, 3.23) | 0.7608 | 1.02 (0.26, 4.09) | 0.9779 | 1.05 (0.26, 4.20) | 0.9455 |
| MAFLD (diabetes) | 18,335 | 224 (1.22) | 2.38 (2.07, 2.74) | <0.0001 | 1.56 (1.35, 1.80) | <0.0001 | 1.46 (1.26, 1.69) | <0.0001 |
| MAFLD (overweight/obesity) | 133,903 | 702 (0.52) | 0.94 (0.86, 1.03) | 0.1937 | 0.84 (0.77, 0.92) | 0.0003 | 0.83 (0.76, 0.91) | <0.0001 |
| MAFLD (lean metabolic disorder) | 2,796 | 16 (0.57) | 1.07 (0.65, 1.74) | 0.8028 | 0.87 (0.53, 1.43) | 0.5832 | 0.87 (0.53, 1.43) | 0.5865 |
| **Vascular dementia** |  |  |  |  |  |  |  |  |
| MAFLD– | 248,397 | 597 (0.24) | Reference | - | Reference | - | Reference | - |
| MAFLD+ | 155,034 | 662 (0.43) | 1.82 (1.63, 2.03) | <0.0001 | 1.42 (1.27, 1.60) | <0.0001 | 1.32 (1.17, 1.48) | <0.0001 |
| No SLD | 247,946 | 596 (0.24) | Reference | - | Reference | - | Reference | - |
| Non-MAFLD steatosis | 451 | 1 (0.22) | 0.95 (0.13, 6.74) | 0.9571 | 1.03 (0.15, 7.33) | 0.976 | 1.14 (0.16, 8.14) | 0.8931 |
| MAFLD (diabetes) | 18,335 | 244 (1.33) | 6.08 (5.24, 7.06) | <0.0001 | 3.58 (3.07, 4.18) | <0.0001 | 2.95 (2.52, 3.45) | <0.0001 |
| MAFLD (overweight/obesity) | 133,903 | 400 (0.30) | 1.26 (1.11, 1.43) | 0.0003 | 1.04 (0.92, 1.19) | 0.5222 | 0.99 (0.87, 1.13) | 0.9246 |
| MAFLD (lean metabolic disorder) | 2,796 | 18 (0.64) | 2.82 (1.76, 4.50) | <0.0001 | 2.02 (1.26, 3.24) | 0.0035 | 2.03 (1.27, 3.25) | 0.0032 |
| **Frontotemporal dementia** |  |  |  |  |  |  |  |  |
| MAFLD– | 248,397 | 110 (0.04) | Reference | - | Reference | - | Reference | - |
| MAFLD+ | 155,034 | 68 (0.04) | 1.01 (0.75, 1.37) | 0.9357 | 0.82 (0.60, 1.12) | 0.216 | 0.81 (0.59, 1.11) | 0.197 |
| No SLD | 247,946 | 110 (0.04) | Reference | - | Reference | - | Reference | - |
| Non-MAFLD steatosis | 451 | 0 (0.00) | - | - | - | - | - | - |
| MAFLD (diabetes) | 18,335 | 9 (0.05) | 1.20 (0.61, 2.37) | 0.6016 | 0.78 (0.39, 1.54) | 0.4675 | 0.75 (0.38, 1.50) | 0.4181 |
| MAFLD (overweight/obesity) | 133,903 | 58 (0.04) | 0.99 (0.72, 1.36) | 0.9507 | 0.83 (0.60, 1.15) | 0.2629 | 0.82 (0.59, 1.15) | 0.2487 |
| MAFLD (lean metabolic disorder) | 2,796 | 1 (0.04) | 0.84 (0.12, 6.05) | 0.8662 | 0.64 (0.09, 4.62) | 0.6596 | 0.64 (0.09, 4.62) | 0.6596 |

HR, hazard ratio; MAFLD, metabolic dysfunction associated fatty liver disease; MASLD, metabolic dysfunction-associated steatotic liver disease; MetALD, MASLD with greater alcohol consumption; SLD, steatotic liver diseases.

^a^ Analysis by Cox proportional hazards model.

^b^ Adjusted for age, sex, ethnicity, education, Townsend deprivation index, income levels, smoking status, alcohol intake and IPAQ activity group.

^c^ Additionally adjusted for *APOE ε4* genotypes and cardiovascular diseases.

# Supplementary Table 7. Association of MASLD and SLD subtypes with all-cause and cause-specific dementia excluding participants with incident dementia within the first 2 years of follow-up.

|  | **No. of participants** | **No. of cases (%)** | **Crude model** | | **Multivariable model 1^b^** | | **Multivariable model 2^c^** | |
| --- | --- | --- | --- | --- | --- | --- | --- | --- |
|  |  |  | **HR (95% CI)** | ***P* value^a^** | **HR (95% CI)** | ***P* value^a^** | **HR (95% CI)** | ***P* value^a^** |
| **All-cause dementia** |  |  |  |  |  |  |  |  |
| MASLD– | 291,516 | 3,756 (1.29) | Reference | - | Reference | - | Reference | - |
| MASLD+ | 111,915 | 1,901 (1.7) | 1.35 (1.27, 1.42) | <0.0001 | 1.09 (1.03, 1.16) | 0.0031 | 1.05 (0.99, 1.11) | 0.0882 |
| No SLD | 247,946 | 3,157 (1.27) | Reference | - | Reference | - | Reference | - |
| MASLD | 111,915 | 1,901 (1.7) | 1.37 (1.29, 1.45) | <0.0001 | 1.10 (1.03, 1.16) | 0.0028 | 1.05 (0.99, 1.11) | 0.1132 |
| MetALD | 43,516 | 598 (1.37) | 1.11 (1.02, 1.21) | 0.0175 | 1.02 (0.93, 1.12) | 0.6417 | 0.99 (0.90, 1.08) | 0.754 |
| Cryptogenic SLD | 30 | 0 (0.00) | - | - | - | - | - | - |
| Other specific etiology SLD | 24 | 1 (4.17) | 4.39 (0.62, 31.2) | 0.1393 | 6.99 (0.98, 49.7) | 0.0519 | 5.28 (0.74, 37.5) | 0.0963 |
| **Alzheimer’s disease** |  |  |  |  |  |  |  |  |
| MASLD– | 291,516 | 1,601 (0.55) | Reference | - | Reference | - | Reference | - |
| MASLD+ | 111,915 | 745 (0.67) | 1.24 (1.13, 1.35) | <0.0001 | 1.02 (0.93, 1.12) | 0.6753 | 1.00 (0.91, 1.09) | 0.9809 |
| No SLD | 247,946 | 1,402 (0.57) | Reference | - | Reference | - | Reference | - |
| MASLD | 111,915 | 745 (0.67) | 1.21 (1.10, 1.32) | <0.0001 | 0.99 (0.9, 1.09) | 0.8354 | 0.97 (0.88, 1.06) | 0.4636 |
| MetALD | 43,516 | 199 (0.46) | 0.83 (0.72, 0.97) | 0.0156 | 0.80 (0.69, 0.93) | 0.0044 | 0.78 (0.67, 0.92) | 0.0019 |
| Cryptogenic SLD | 30 | 0 (0.00) | - | - | - | - | - | - |
| Other specific etiology SLD | 24 | 0 (0.00) | - | - | - | - | - | - |
| **Vascular dementia** |  |  |  |  |  |  |  |  |
| MASLD– | 291,516 | 764 (0.26) | Reference | - | Reference | - | Reference | - |
| MASLD+ | 111,915 | 495 (0.44) | 1.72 (1.54, 1.93) | <0.0001 | 1.34 (1.19, 1.50) | <0.0001 | 1.24 (1.10, 1.39) | 0.0004 |
| No SLD | 247,946 | 596 (0.24) | Reference | - | Reference | - | Reference | - |
| MASLD | 111,915 | 495 (0.44) | 1.88 (1.67, 2.12) | <0.0001 | 1.43 (1.26, 1.61) | <0.0001 | 1.31 (1.16, 1.49) | <0.0001 |
| MetALD | 43,516 | 167 (0.38) | 1.64 (1.38, 1.95) | <0.0001 | 1.41 (1.17, 1.69) | 0.0002 | 1.32 (1.11, 1.58) | 0.0022 |
| Cryptogenic SLD | 30 | 0 (0.00) | - | - | - | - | - | - |
| Other specific etiology SLD | 24 | 1 (4.17) | 23.1 (3.3, 164.1) | 0.0017 | 36.6 (5.1, 260.7) | 0.0003 | 21.9 (3.1, 156.4) | 0.0021 |
| **Frontotemporal dementia** |  |  |  |  |  |  |  |  |
| MASLD– | 291,516 | 129 (0.04) | Reference | - | Reference | - | Reference | - |
| MASLD+ | 111,915 | 49 (0.04) | 1.01 (0.72, 1.40) | 0.974 | 0.77 (0.54, 1.08) | 0.1337 | 0.76 (0.54, 1.08) | 0.1234 |
| No SLD | 247,946 | 110 (0.04) | Reference | - | Reference | - | Reference | - |
| MASLD | 111,915 | 49 (0.04) | 1.01 (0.72, 1.41) | 0.9679 | 0.77 (0.54, 1.09) | 0.137 | 0.76 (0.54, 1.08) | 0.1251 |
| MetALD | 43,516 | 19 (0.04) | 1.01 (0.62, 1.65) | 0.9644 | 0.98 (0.58, 1.66) | 0.9478 | 0.97 (0.58, 1.64) | 0.9202 |
| Cryptogenic SLD | 30 | 0 (0.00) | - | - | - | - | - | - |
| Other specific etiology SLD | 24 | 0 (0.00) | - | - | - | - | - | - |

HR, hazard ratio; MAFLD, metabolic dysfunction associated fatty liver disease; MASLD, metabolic dysfunction-associated steatotic liver disease; MetALD, MASLD with greater alcohol consumption; SLD, steatotic liver diseases.

^a^ Analysis by Cox proportional hazards model.

^b^ Adjusted for age, sex, ethnicity, education, Townsend deprivation index, income levels, smoking status, alcohol intake and IPAQ activity group.

^c^ Additionally adjusted for *APOE ε4* genotypes and cardiovascular diseases.

# Supplementary Table 8. Risk of all-cause and cause-specific dementia according to presence and combination of MAFLD and/or MASLD excluding participants with incident dementia within the first 2 years of follow-up.

|  | **No. of participants** | **No. of cases (%)** | **Crude model** | | **Multivariable model 1^b^** | | **Multivariable model 2^c^** | |
| --- | --- | --- | --- | --- | --- | --- | --- | --- |
|  |  |  | **HR (95% CI)** | ***P* value^a^** | **HR (95% CI)** | ***P* value^a^** | **HR (95% CI)** | ***P* value^a^** |
| **All-cause dementia** |  |  |  |  |  |  |  |  |
| MAFLD–/MASLD– | 248,172 | 3,161 (1.27) | Reference | - | Reference | - | Reference | - |
| MAFLD+/MASLD– | 43,344 | 595 (1.37) | 1.11 (1.02, 1.21) | 0.0195 | 1.02 (0.93, 1.12) | 0.6943 | 0.98 (0.90, 1.08) | 0.6987 |
| MAFLD–/MASLD+ | 225 | 0 (0.00) | - | - | - | - | - | - |
| MAFLD+/MASLD+ | 111,690 | 1,901 (1.7) | 1.37 (1.29, 1.45) | <0.0001 | 1.10 (1.03, 1.16) | 0.0026 | 1.05 (0.99, 1.11) | 0.1085 |
| **Alzheimer’s disease** |  |  |  |  |  |  |  |  |
| MAFLD–/MASLD– | 248,172 | 1,404 (0.57) | Reference | - | Reference | - | Reference | - |
| MAFLD+/MASLD– | 43,344 | 197 (0.45) | 0.83 (0.71, 0.96) | 0.0125 | 0.79 (0.68, 0.93) | 0.0032 | 0.78 (0.67, 0.91) | 0.0014 |
| MAFLD–/MASLD+ | 225 | 0 (0.00) | - | - | - | - | - | - |
| MAFLD+/MASLD+ | 111,690 | 745 (0.67) | 1.21 (1.11, 1.32) | <0.0001 | 0.99 (0.90, 1.09) | 0.8441 | 0.97 (0.88, 1.06) | 0.4691 |
| **Vascular dementia** |  |  |  |  |  |  |  |  |
| MAFLD–/MASLD– | 248,172 | 597 (0.24) | Reference | - | Reference | - | Reference | - |
| MAFLD+/MASLD– | 43,344 | 167 (0.39) | 1.65 (1.39, 1.96) | <0.0001 | 1.41 (1.18, 1.69) | 0.0002 | 1.32 (1.11, 1.58) | 0.0021 |
| MAFLD–/MASLD+ | 225 | 0 (0.00) | - | - | - | - | - | - |
| MAFLD+/MASLD+ | 111,690 | 495 (0.44) | 1.89 (1.67, 2.12) | <0.0001 | 1.43 (1.26, 1.61) | <0.0001 | 1.31 (1.16, 1.48) | <0.0001 |
| **Frontotemporal dementia** |  |  |  |  |  |  |  |  |
| MAFLD–/MASLD– | 248,172 | 110 (0.04) | Reference | - | Reference | - | Reference | - |
| MAFLD+/MASLD– | 43,344 | 19 (0.04) | 1.02 (0.62, 1.65) | 0.949 | 0.99 (0.59, 1.66) | 0.9625 | 0.98 (0.58, 1.65) | 0.9348 |
| MAFLD–/MASLD+ | 225 | 0 (0.00) | - | - | - | - | - | - |
| MAFLD+/MASLD+ | 111,690 | 49 (0.04) | 1.01 (0.72, 1.41) | 0.9544 | 0.77 (0.54, 1.09) | 0.1408 | 0.76 (0.54, 1.08) | 0.1287 |

HR, hazard ratio; MAFLD, metabolic dysfunction associated fatty liver disease; MASLD, metabolic dysfunction-associated steatotic liver disease; MetALD, MASLD with greater alcohol consumption; SLD, steatotic liver diseases.

^a^ Analysis by Cox proportional hazards model.

^b^ Adjusted for age, sex, ethnicity, education, Townsend deprivation index, income levels, smoking status, alcohol intake and IPAQ activity group.

^c^ Additionally adjusted for *APOE ε4* genotypes and cardiovascular diseases.

# Supplementary Table 9. Association of MAFLD and its subtypes with all-cause and cause-specific dementia excluding participants with incident dementia within the first 5 years of follow-up.

|  | **No. of participants** | **No. of cases (%)** | **Crude model** | | **Multivariable model 1^b^** | | **Multivariable model 2^c^** | |
| --- | --- | --- | --- | --- | --- | --- | --- | --- |
|  |  |  | **HR (95% CI)** | ***P* value^a^** | **HR (95% CI)** | ***P* value^a^** | **HR (95% CI)** | ***P* value^a^** |
| **All-cause dementia** |  |  |  |  |  |  |  |  |
| MAFLD– | 248,190 | 2,954 (1.19) | Reference | - | Reference | - | Reference | - |
| MAFLD+ | 154,862 | 2,324 (1.50) | 1.30 (1.23, 1.37) | <0.0001 | 1.08 (1.02, 1.14) | 0.0096 | 1.04 (0.98, 1.10) | 0.2226 |
| No SLD | 247,739 | 2,950 (1.19) | Reference | - | Reference | - | Reference | - |
| Non-MAFLD steatosis | 451 | 4 (0.89) | 0.77 (0.29, 2.05) | 0.599 | 0.91 (0.34, 2.43) | 0.8491 | 0.95 (0.36, 2.54) | 0.9214 |
| MAFLD (diabetes) | 18,284 | 611 (3.34) | 3.12 (2.86, 3.40) | <0.0001 | 2.01 (1.84, 2.20) | <0.0001 | 1.81 (1.65, 1.98) | <0.0001 |
| MAFLD (overweight/obesity) | 133,788 | 1,666 (1.25) | 1.06 (1, 1.13) | 0.045 | 0.93 (0.87, 0.98) | 0.013 | 0.90 (0.85, 0.96) | 0.0011 |
| MAFLD (lean metabolic disorder) | 2,790 | 47 (1.68) | 1.49 (1.12, 1.99) | 0.0064 | 1.17 (0.88, 1.56) | 0.2906 | 1.17 (0.88, 1.56) | 0.2845 |
| **Alzheimer’s disease** |  |  |  |  |  |  |  |  |
| MAFLD– | 248,190 | 1,315 (0.53) | Reference | - | Reference | - | Reference | - |
| MAFLD+ | 154,862 | 890 (0.57) | 1.11 (1.02, 1.21) | 0.0131 | 0.96 (0.88, 1.04) | 0.3204 | 0.94 (0.86, 1.02) | 0.1339 |
| No SLD | 247,739 | 1,313 (0.53) | Reference | - | Reference | - | Reference | - |
| Non-MAFLD steatosis | 451 | 2 (0.44) | 0.86 (0.22, 3.45) | 0.8339 | 1.10 (0.27, 4.40) | 0.8946 | 1.13 (0.28, 4.52) | 0.8639 |
| MAFLD (diabetes) | 18,284 | 213 (1.16) | 2.43 (2.10, 2.81) | <0.0001 | 1.60 (1.38, 1.86) | <0.0001 | 1.51 (1.30, 1.75) | <0.0001 |
| MAFLD (overweight/obesity) | 133,788 | 662 (0.49) | 0.95 (0.86, 1.04) | 0.2719 | 0.85 (0.77, 0.94) | 0.001 | 0.84 (0.76, 0.92) | 0.0003 |
| MAFLD (lean metabolic disorder) | 2,790 | 15 (0.54) | 1.07 (0.64, 1.78) | 0.7977 | 0.88 (0.53, 1.46) | 0.613 | 0.88 (0.53, 1.46) | 0.6168 |
| **Vascular dementia** |  |  |  |  |  |  |  |  |
| MAFLD– | 248,190 | 551 (0.22) | Reference | - | Reference | - | Reference | - |
| MAFLD+ | 154,862 | 616 (0.40) | 1.84 (1.64, 2.06) | <0.0001 | 1.46 (1.30, 1.65) | <0.0001 | 1.35 (1.20, 1.53) | <0.0001 |
| No SLD | 247,739 | 550 (0.22) | Reference | - | Reference | - | Reference | - |
| Non-MAFLD steatosis | 451 | 1 (0.22) | 1.03 (0.15, 7.31) | 0.9777 | 1.15 (0.16, 8.21) | 0.8866 | 1.28 (0.18, 9.10) | 0.8065 |
| MAFLD (diabetes) | 18,284 | 225 (1.23) | 6.11 (5.23, 7.14) | <0.0001 | 3.68 (3.13, 4.32) | <0.0001 | 3.04 (2.58, 3.58) | <0.0001 |
| MAFLD (overweight/obesity) | 133,788 | 374 (0.28) | 1.28 (1.12, 1.46) | 0.0002 | 1.07 (0.94, 1.23) | 0.2989 | 1.02 (0.89, 1.17) | 0.7324 |
| MAFLD (lean metabolic disorder) | 2,790 | 17 (0.61) | 2.89 (1.78, 4.68) | <0.0001 | 2.12 (1.31, 3.45) | 0.0024 | 2.13 (1.31, 3.47) | 0.0022 |
| **Frontotemporal dementia** |  |  |  |  |  |  |  |  |
| MAFLD– | 248,190 | 98 (0.04) | Reference | - | Reference | - | Reference | - |
| MAFLD+ | 154,862 | 61 (0.04) | 1.02 (0.74, 1.41) | 0.8957 | 0.82 (0.59, 1.15) | 0.2482 | 0.81 (0.58, 1.14) | 0.2266 |
| No SLD | 247,739 | 98 (0.04) | Reference | - | Reference | - | Reference | - |
| Non-MAFLD steatosis | 451 | 0 (0.00) | - | - | - | - | - | - |
| MAFLD (diabetes) | 18,284 | 9 (0.05) | 1.36 (0.69, 2.68) | 0.3823 | 0.87 (0.44, 1.74) | 0.6952 | 0.84 (0.42, 1.70) | 0.6309 |
| MAFLD (overweight/obesity) | 133,788 | 51 (0.04) | 0.98 (0.70, 1.37) | 0.8991 | 0.81 (0.57, 1.16) | 0.2493 | 0.81 (0.57, 1.15) | 0.2358 |
| MAFLD (lean metabolic disorder) | 2,790 | 1 (0.04) | 0.95 (0.13, 6.83) | 0.9608 | 0.72 (0.10, 5.20) | 0.7465 | 0.72 (0.10, 5.21) | 0.7466 |

HR, hazard ratio; MAFLD, metabolic dysfunction associated fatty liver disease; MASLD, metabolic dysfunction-associated steatotic liver disease; MetALD, MASLD with greater alcohol consumption; SLD, steatotic liver diseases.

^a^ Analysis by Cox proportional hazards model.

^b^ Adjusted for age, sex, ethnicity, education, Townsend deprivation index, income levels, smoking status, alcohol intake and IPAQ activity group.

^c^ Additionally adjusted for *APOE ε4* genotypes and cardiovascular diseases.

# Supplementary Table 10. Association of MASLD and SLD subtypes with all-cause and cause-specific dementia excluding participants with incident dementia within the first 5 years of follow-up.

|  | **No. of participants** | **No. of cases (%)** | **Crude model** | | **Multivariable model 1^b^** | | **Multivariable model 2^c^** | |
| --- | --- | --- | --- | --- | --- | --- | --- | --- |
|  |  |  | **HR (95% CI)** | ***P* value^a^** | **HR (95% CI)** | ***P* value^a^** | **HR (95% CI)** | ***P* value^a^** |
| **All-cause dementia** |  |  |  |  |  |  |  |  |
| MASLD– | 291,260 | 3,500 (1.20) | Reference | - | Reference | - | Reference | - |
| MASLD+ | 111,792 | 1,778 (1.59) | 1.35 (1.28, 1.43) | <0.0001 | 1.10 (1.03, 1.17) | 0.0021 | 1.06 (1, 1.13) | 0.0557 |
| No SLD | 247,739 | 2,950 (1.19) | Reference | - | Reference | - | Reference | - |
| MASLD | 111,792 | 1,778 (1.59) | 1.37 (1.29, 1.45) | <0.0001 | 1.10 (1.03, 1.17) | 0.0024 | 1.06 (0.99, 1.12) | 0.0828 |
| MetALD | 43,467 | 549 (1.26) | 1.09 (1.00, 1.20) | 0.0533 | 1.01 (0.92, 1.11) | 0.8585 | 0.98 (0.89, 1.07) | 0.5952 |
| Cryptogenic SLD | 30 | 0 (0.00) | - | - | - | - | - | - |
| Other specific etiology SLD | 24 | 1 (4.17) | 4.78 (0.67, 33.9) | 0.118 | 7.59 (1.07, 53.9) | 0.0428 | 5.73 (0.81, 40.8) | 0.081 |
| **Alzheimer’s disease** |  |  |  |  |  |  |  |  |
| MASLD– | 291,260 | 1,500 (0.52) | Reference | - | Reference | - | Reference | - |
| MASLD+ | 111,792 | 705 (0.63) | 1.25 (1.14, 1.37) | <0.0001 | 1.04 (0.95, 1.14) | 0.3977 | 1.02 (0.93, 1.12) | 0.6865 |
| No SLD | 247,739 | 1,313 (0.53) | Reference | - | Reference | - | Reference | - |
| MASLD | 111,792 | 705 (0.63) | 1.22 (1.11, 1.34) | <0.0001 | 1.01 (0.92, 1.11) | 0.8552 | 0.98 (0.90, 1.08) | 0.7414 |
| MetALD | 43,467 | 187 (0.43) | 0.84 (0.72, 0.98) | 0.0225 | 0.80 (0.68, 0.94) | 0.0053 | 0.79 (0.67, 0.92) | 0.0027 |
| Cryptogenic SLD | 30 | 0 (0.00) | - | - | - | - | - | - |
| Other specific etiology SLD | 24 | 0 (0.00) | - | - | - | - | - | - |
| **Vascular dementia** |  |  |  |  |  |  |  |  |
| MASLD– | 291,260 | 704 (0.24) | Reference | - | Reference | - | Reference | - |
| MASLD+ | 111,792 | 463 (0.41) | 1.75 (1.56, 1.97) | <0.0001 | 1.38 (1.22, 1.55) | <0.0001 | 1.27 (1.13, 1.44) | <0.0001 |
| No SLD | 247,739 | 550 (0.22) | Reference | - | Reference | - | Reference | - |
| MASLD | 111,792 | 463 (0.41) | 1.91 (1.69, 2.16) | <0.0001 | 1.47 (1.30, 1.68) | <0.0001 | 1.36 (1.19, 1.54) | <0.0001 |
| MetALD | 43,467 | 153 (0.35) | 1.63 (1.37, 1.96) | <0.0001 | 1.41 (1.17, 1.70) | 0.0003 | 1.34 (1.11, 1.61) | 0.0021 |
| Cryptogenic SLD | 30 | 0 (0.00) | - | - | - | - | - | - |
| Other specific etiology SLD | 24 | 1 (4.17) | 25.4 (3.58, 180.9) | 0.0012 | 40.09 (5.62, 285.9) | 0.0002 | 23.96 (3.35, 171.1) | 0.0015 |
| **Frontotemporal dementia** |  |  |  |  |  |  |  |  |
| MASLD– | 291,260 | 117 (0.04) | Reference | - | Reference | - | Reference | - |
| MASLD+ | 111,792 | 42 (0.04) | 0.95 (0.67, 1.36) | 0.7855 | 0.73 (0.50, 1.05) | 0.0879 | 0.72 (0.50, 1.04) | 0.08 |
| No SLD | 247,739 | 98 (0.04) | Reference | - | Reference | - | Reference | - |
| MASLD | 111,792 | 42 (0.04) | 0.97 (0.68, 1.39) | 0.8715 | 0.73 (0.50, 1.07) | 0.1073 | 0.73 (0.50, 1.06) | 0.0972 |
| MetALD | 43,467 | 19 (0.04) | 1.14 (0.70, 1.86) | 0.6069 | 1.10 (0.65, 1.87) | 0.7251 | 1.09 (0.64, 1.85) | 0.7525 |
| Cryptogenic SLD | 30 | 0 (0.00) | - | - | - | - | - | - |
| Other specific etiology SLD | 24 | 0 (0.00) | - | - | - | - | - | - |

HR, hazard ratio; MAFLD, metabolic dysfunction associated fatty liver disease; MASLD, metabolic dysfunction-associated steatotic liver disease; MetALD, MASLD with greater alcohol consumption; SLD, steatotic liver diseases.

^a^ Analysis by Cox proportional hazards model.

^b^ Adjusted for age, sex, ethnicity, education, Townsend deprivation index, income levels, smoking status, alcohol intake and IPAQ activity group.

^c^ Additionally adjusted for *APOE ε4* genotypes and cardiovascular diseases.

# Supplementary Table 11. Risk of all-cause and cause-specific dementia according to presence and combination of MAFLD and/or MASLD excluding participants with incident dementia within the first 5 years of follow-up.

|  | **No. of participants** | **No. of cases (%)** | **Crude model** | | **Multivariable model 1^b^** | | **Multivariable model 2^c^** | |
| --- | --- | --- | --- | --- | --- | --- | --- | --- |
|  |  |  | **HR (95% CI)** | ***P* value^a^** | **HR (95% CI)** | ***P* value^a^** | **HR (95% CI)** | ***P* value^a^** |
| **All-cause dementia** |  |  |  |  |  |  |  |  |
| MAFLD–/MASLD– | 247,965 | 2,954 (1.19) | Reference | - | Reference | - | Reference | - |
| MAFLD+/MASLD– | 43,295 | 546 (1.26) | 1.09 (1.00, 1.20) | 0.0597 | 1.01 (0.91, 1.11) | 0.9236 | 0.97 (0.88, 1.07) | 0.5388 |
| MAFLD–/MASLD+ | 225 | 0 (0.00) | - | - | - | - | - | - |
| MAFLD+/MASLD+ | 111,567 | 1,778 (1.59) | 1.37 (1.29, 1.46) | <0.0001 | 1.10 (1.04, 1.17) | 0.0022 | 1.06 (0.99, 1.12) | 0.0799 |
| **Alzheimer’s disease** |  |  |  |  |  |  |  |  |
| MAFLD–/MASLD– | 247,965 | 1,315 (0.53) | Reference | - | Reference | - | Reference | - |
| MAFLD+/MASLD– | 43,295 | 185 (0.43) | 0.83 (0.71, 0.97) | 0.0179 | 0.79 (0.68, 0.93) | 0.0038 | 0.78 (0.66, 0.91) | 0.0019 |
| MAFLD–/MASLD+ | 225 | 0 (0.00) | - | - | - | - | - | - |
| MAFLD+/MASLD+ | 111,567 | 705 (0.63) | 1.22 (1.12, 1.34) | <0.0001 | 1.01 (0.92, 1.11) | 0.8491 | 0.98 (0.9, 1.08) | 0.7458 |
| **Vascular dementia** |  |  |  |  |  |  |  |  |
| MAFLD–/MASLD– | 247,965 | 551 (0.22) | Reference | - | Reference | - | Reference | - |
| MAFLD+/MASLD– | 43,295 | 153 (0.35) | 1.64 (1.37, 1.96) | <0.0001 | 1.42 (1.17, 1.71) | 0.0003 | 1.34 (1.11, 1.61) | 0.0021 |
| MAFLD–/MASLD+ | 225 | 0 (0.00) | - | - | - | - | - | - |
| MAFLD+/MASLD+ | 111,567 | 463 (0.41) | 1.91 (1.69, 2.17) | <0.0001 | 1.47 (1.30, 1.68) | <0.0001 | 1.36 (1.19, 1.54) | <0.0001 |
| **Frontotemporal dementia** |  |  |  |  |  |  |  |  |
| MAFLD–/MASLD– | 247,965 | 98 (0.04) | Reference | - | Reference | - | Reference | - |
| MAFLD+/MASLD– | 43,295 | 19 (0.04) | 1.14 (0.70, 1.87) | 0.5936 | 1.11 (0.65, 1.88) | 0.7117 | 1.10 (0.64, 1.86) | 0.739 |
| MAFLD–/MASLD+ | 225 | 0 (0.00) | - | - | - | - | - | - |
| MAFLD+/MASLD+ | 111,567 | 42 (0.04) | 0.97 (0.68, 1.40) | 0.884 | 0.74 (0.51, 1.07) | 0.1102 | 0.73 (0.50, 1.06) | 0.0999 |

HR, hazard ratio; MAFLD, metabolic dysfunction associated fatty liver disease; MASLD, metabolic dysfunction-associated steatotic liver disease; MetALD, MASLD with greater alcohol consumption; SLD, steatotic liver diseases.

^a^ Analysis by Cox proportional hazards model.

^b^ Adjusted for age, sex, ethnicity, education, Townsend deprivation index, income levels, smoking status, alcohol intake and IPAQ activity group.

^c^ Additionally adjusted for *APOE ε4* genotypes and cardiovascular diseases.

# Supplementary Table 12. Association of MAFLD and its subtypes with all-cause and cause-specific dementia taking account competing risk of death from other causes.

|  | **No. of participants** | **No. of cases (%)** | **Crude model** | | **Multivariable model 1^b^** | | **Multivariable model 2^c^** | |
| --- | --- | --- | --- | --- | --- | --- | --- | --- |
|  |  |  | **HR (95% CI)** | ***P* value^a^** | **HR (95% CI)** | ***P* value^a^** | **HR (95% CI)** | ***P* value^a^** |
| **All-cause dementia** |  |  |  |  |  |  |  |  |
| MAFLD– | 248,438 | 3,202 (1.29) | Reference | - | Reference | - | Reference | - |
| MAFLD+ | 155,068 | 2,530 (1.63) | 1.27 (1.20, 1.34) | <0.0001 | 1.05 (1.00, 1.12) | 0.0652 | 1.01 (0.96, 1.07) | 0.6391 |
| No SLD | 247,986 | 3,197 (1.29) | Reference | - | Reference | - | Reference | - |
| Non-MAFLD steatosis | 452 | 5 (1.11) | 0.86 (0.36, 2.07) | 0.7356 | 1.00 (0.41, 2.43) | 0.9964 | 1.04 (0.43, 2.52) | 0.9354 |
| MAFLD (diabetes) | 18,345 | 672 (3.66) | 2.88 (2.65, 3.13) | <0.0001 | 1.85 (1.69, 2.03) | <0.0001 | 1.66 (1.52, 1.81) | <0.0001 |
| MAFLD (overweight/obesity) | 133,927 | 1,805 (1.35) | 1.05 (0.99, 1.11) | 0.1299 | 0.91 (0.86, 0.97) | 0.0027 | 0.89 (0.84, 0.95) | 0.0002 |
| MAFLD (lean metabolic disorder) | 2,796 | 53 (1.90) | 1.47 (1.12, 1.93) | 0.005 | 1.17 (0.89, 1.54) | 0.2505 | 1.17 (0.89, 1.54) | 0.2659 |
| **Alzheimer’s disease** |  |  |  |  |  |  |  |  |
| MAFLD– | 248,438 | 1,411 (0.57) | Reference | - | Reference | - | Reference | - |
| MAFLD+ | 155,068 | 944 (0.61) | 1.07 (0.99, 1.17) | 0.0958 | 0.92 (0.84, 1.00) | 0.0603 | 0.90 (0.83, 0.98) | 0.0188 |
| No SLD | 247,986 | 1,409 (0.57) | Reference | - | Reference | - | Reference | - |
| Non-MAFLD steatosis | 452 | 2 (0.44) | 0.78 (0.19, 3.12) | 0.7238 | 0.99 (0.24, 3.98) | 0.9832 | 1.01 (0.25, 4.06) | 0.9935 |
| MAFLD (diabetes) | 18,345 | 225 (1.23) | 2.17 (1.88, 2.49) | <0.0001 | 1.41 (1.22, 1.64) | <0.0001 | 1.34 (1.15, 1.55) | 0.0001 |
| MAFLD (overweight/obesity) | 133,927 | 703 (0.52) | 0.92 (0.84, 1.01) | 0.0851 | 0.83 (0.76, 0.91) | 0.0001 | 0.82 (0.75, 0.90) | <0.0001 |
| MAFLD (lean metabolic disorder) | 2,796 | 16 (0.57) | 1.01 (0.62, 1.65) | 0.977 | 0.84 (0.51, 1.39) | 0.502 | 0.84 (0.51, 1.38) | 0.4969 |
| **Vascular dementia** |  |  |  |  |  |  |  |  |
| MAFLD– | 248,438 | 602 (0.24) | Reference | - | Reference | - | Reference | - |
| MAFLD+ | 155,068 | 672 (0.43) | 1.79 (1.60, 2.00) | <0.0001 | 1.40 (1.25, 1.58) | <0.0001 | 1.30 (1.16, 1.46) | <0.0001 |
| No SLD | 247,986 | 601 (0.24) | Reference | - | Reference | - | Reference | - |
| Non-MAFLD steatosis | 452 | 1 (0.22) | 0.91 (0.13, 6.51) | 0.928 | 1.00 (0.14, 7.14) | 0.9976 | 1.09 (0.15, 7.78) | 0.9348 |
| MAFLD (diabetes) | 18,345 | 248 (1.35) | 5.61 (4.84, 6.51) | <0.0001 | 3.31 (2.80, 3.91) | <0.0001 | 2.72 (2.3, 3.21) | <0.0001 |
| MAFLD (overweight/obesity) | 133,927 | 406 (0.30) | 1.25 (1.10, 1.42) | 0.0005 | 1.04 (0.92, 1.19) | 0.5319 | 1.00 (0.88, 1.14) | 0.9753 |
| MAFLD (lean metabolic disorder) | 2,796 | 18 (0.64) | 2.66 (1.67, 4.25) | <0.0001 | 1.96 (1.22, 3.15) | 0.0052 | 1.95 (1.22, 3.13) | 0.0056 |
| **Frontotemporal dementia** |  |  |  |  |  |  |  |  |
| MAFLD– | 248,438 | 110 (0.04) | Reference | - | Reference | - | Reference | - |
| MAFLD+ | 155,068 | 70 (0.05) | 1.02 (0.76, 1.38) | 0.8992 | 0.83 (0.61, 1.14) | 0.2574 | 0.83 (0.60, 1.14) | 0.2471 |
| No SLD | 247,986 | 110 (0.04) | Reference | - | Reference | - | Reference | - |
| Non-MAFLD steatosis | 452 | 0 (0.00) | - | - | - | - | - | - |
| MAFLD (diabetes) | 18,345 | 11 (0.06) | 1.35 (0.73, 2.51) | 0.3404 | 0.88 (0.47, 1.64) | 0.6811 | 0.86 (0.46, 1.62) | 0.645 |
| MAFLD (overweight/obesity) | 133,927 | 58 (0.04) | 0.98 (0.71, 1.34) | 0.8825 | 0.83 (0.59, 1.15) | 0.2628 | 0.82 (0.59, 1.15) | 0.257 |
| MAFLD (lean metabolic disorder) | 2,796 | 1 (0.04) | 0.81 (0.11, 5.77) | 0.8302 | 0.63 (0.09, 4.63) | 0.6499 | 0.63 (0.09, 4.62) | 0.6494 |

HR, hazard ratio; MAFLD, metabolic dysfunction associated fatty liver disease; MASLD, metabolic dysfunction-associated steatotic liver disease; MetALD, MASLD with greater alcohol consumption; SLD, steatotic liver diseases.

^a^ Analysis by Cox proportional hazards model.

^b^ Adjusted for age, sex, ethnicity, education, Townsend deprivation index, income levels, smoking status, alcohol intake and IPAQ activity group.

^c^ Additionally adjusted for *APOE ε4* genotypes and cardiovascular diseases.

# Supplementary Table 13. Association of MASLD and SLD subtypes with all-cause and cause-specific dementia taking account competing risk of death from other causes.

|  | **No. of participants** | **No. of cases (%)** | **Crude model** | | **Multivariable model 1^b^** | | **Multivariable model 2^c^** | |
| --- | --- | --- | --- | --- | --- | --- | --- | --- |
|  |  |  | **HR (95% CI)** | ***P* value^a^** | **HR (95% CI)** | ***P* value^a^** | **HR (95% CI)** | ***P* value^a^** |
| **All-cause dementia** |  |  |  |  |  |  |  |  |
| MASLD– | 291,568 | 3,808 (1.31) | Reference | - | Reference | - | Reference | - |
| MASLD+ | 111,938 | 1,924 (1.72) | 1.32 (1.25, 1.39) | <0.0001 | 1.08 (1.00, 1.15) | 0.0414 | 1.04 (0.97, 1.11) | 0.2574 |
| No SLD | 247,986 | 3,197 (1.29) | Reference | - | Reference | - | Reference | - |
| MASLD | 111,938 | 1,924 (1.72) | 1.34 (1.26, 1.41) | <0.0001 | 1.08 (1.01, 1.15) | 0.0365 | 1.03 (0.97, 1.10) | 0.3408 |
| MetALD | 43,528 | 610 (1.4) | 1.09 (1.00, 1.19) | 0.0572 | 0.99 (0.90 1.10) | 0.8955 | 0.96 (0.87, 1.06) | 0.4106 |
| Cryptogenic SLD | 30 | 0 (0.00) | - | - | - | - | - | - |
| Other specific etiology SLD | 24 | 1 (4.17) | 3.33 (0.46, 24.4) | 0.2361 | 5.75 (0.74, 44.8) | 0.0949 | 4.81 (0.58, 40.0) | 0.1465 |
| **Alzheimer’s disease** |  |  |  |  |  |  |  |  |
| MASLD– | 291,568 | 1,609 (0.55) | Reference | - | Reference | - | Reference | - |
| MASLD+ | 111,938 | 746 (0.67) | 1.21 (1.11, 1.32) | <0.0001 | 1.00 (0.90, 1.12) | 0.9403 | 0.99 (0.89, 1.10) | 0.7955 |
| No SLD | 247,986 | 1,409 (0.57) | Reference | - | Reference | - | Reference | - |
| MASLD | 111,938 | 746 (0.67) | 1.17 (1.07, 1.28) | 0.0004 | 0.97 (0.88, 1.07) | 0.5333 | 0.95 (0.86, 1.04) | 0.2796 |
| MetALD | 43,528 | 200 (0.46) | 0.81 (0.70, 0.94) | 0.0048 | 0.77 (0.66, 0.91) | 0.0017 | 0.76 (0.65, 0.89) | 0.0007 |
| Cryptogenic SLD | 30 | 0 (0.00) | - | - | - | - | - | - |
| Other specific etiology SLD | 24 | 0 (0.00) | - | - | - | - | - | - |
| **Vascular dementia** |  |  |  |  |  |  |  |  |
| MASLD– | 291,568 | 772 (0.26) | Reference | - | Reference | - | Reference | - |
| MASLD+ | 111,938 | 502 (0.45) | 1.70 (1.52, 1.90) | <0.0001 | 1.33 (1.17, 1.51) | <0.0001 | 1.23 (1.09, 1.39) | 0.0009 |
| No SLD | 247,986 | 601 (0.24) | Reference | - | Reference | - | Reference | - |
| MASLD | 111,938 | 502 (0.45) | 1.85 (1.65, 2.09) | <0.0001 | 1.41 (1.23, 1.62) | <0.0001 | 1.30 (1.14, 1.48) | <0.0001 |
| MetALD | 43,528 | 170 (0.39) | 1.61 (1.36, 1.91) | <0.0001 | 1.37 (1.11, 1.70) | 0.0034 | 1.30 (1.08, 1.56) | 0.0064 |
| Cryptogenic SLD | 30 | 0 (0.00) | - | - | - | - | - | - |
| Other specific etiology SLD | 24 | 1 (4.17) | 17.8 (2.43, 130.3) | 0.0046 | 30.9 (3.83, 248.3) | 0.0013 | 21.8 (2.33, 202.96) | 0.0069 |
| **Frontotemporal dementia** |  |  |  |  |  |  |  |  |
| MASLD– | 291,568 | 129 (0.04) | Reference | - | Reference | - | Reference | - |
| MASLD+ | 111,938 | 51 (0.05) | 1.03 (0.75, 1.42) | 0.8583 | 0.79 (0.56, 1.11) | 0.1686 | 0.79 (0.56, 1.10) | 0.1628 |
| No SLD | 247,986 | 110 (0.04) | Reference | - | Reference | - | Reference | - |
| MASLD | 111,938 | 51 (0.05) | 1.03 (0.74, 1.43) | 0.8745 | 0.79 (0.56, 1.11) | 0.1734 | 0.78 (0.55, 1.11) | 0.1668 |
| MetALD | 43,528 | 19 (0.04) | 0.98 (0.61, 1.60) | 0.9484 | 0.97 (0.57, 1.64) | 0.9064 | 0.96 (0.57, 1.63) | 0.8902 |
| Cryptogenic SLD | 30 | 0 (0.00) | - | - | - | - | - | - |
| Other specific etiology SLD | 24 | 0 (0.00) | - | - | - | - | - | - |

HR, hazard ratio; MAFLD, metabolic dysfunction associated fatty liver disease; MASLD, metabolic dysfunction-associated steatotic liver disease; MetALD, MASLD with greater alcohol consumption; SLD, steatotic liver diseases.

^a^ Analysis by Cox proportional hazards model.

^b^ Adjusted for age, sex, ethnicity, education, Townsend deprivation index, income levels, smoking status, alcohol intake and IPAQ activity group.

^c^ Additionally adjusted for *APOE ε4* genotypes and cardiovascular diseases.

# Supplementary Table 14. Risk of all-cause and cause-specific dementia according to presence and combination of MAFLD and/or MASLD taking account competing risk of death from other causes.

|  | **No. of participants** | **No. of cases (%)** | **Crude model** | | **Multivariable model 1^b^** | | **Multivariable model 2^c^** | |
| --- | --- | --- | --- | --- | --- | --- | --- | --- |
|  |  |  | **HR (95% CI)** | ***P* value^a^** | **HR (95% CI)** | ***P* value^a^** | **HR (95% CI)** | ***P* value^a^** |
| **All-cause dementia** |  |  |  |  |  |  |  |  |
| MAFLD–/MASLD– | 248,213 | 3,202 (1.29) | Reference | - | Reference | - | Reference | - |
| MAFLD+/MASLD– | 43,355 | 606 (1.40) | 1.08 (0.99, 1.18) | 0.0684 | 0.99 (0.89, 1.10) | 0.8187 | 0.96 (0.87, 1.05) | 0.3534 |
| MAFLD–/MASLD+ | 225 | 0 (0.00) | - | - | - | - | - | - |
| MAFLD+/MASLD+ | 111,713 | 1,924 (1.72) | 1.34 (1.26, 1.42) | <0.0001 | 1.08 (1.01, 1.15) | 0.0348 | 1.03 (0.97, 1.10) | 0.3347 |
| **Alzheimer’s disease** |  |  |  |  |  |  |  |  |
| MAFLD–/MASLD– | 248,213 | 1,411 (0.57) | Reference | - | Reference | - | Reference | - |
| MAFLD+/MASLD– | 43,355 | 198 (0.46) | 0.80 (0.69, 0.93) | 0.0038 | 0.76 (0.65, 0.90) | 0.0012 | 0.75 (0.64, 0.88) | 0.0005 |
| MAFLD–/MASLD+ | 225 | 0 (0.00) | - | - | - | - | - | - |
| MAFLD+/MASLD+ | 111,713 | 746 (0.67) | 1.18 (1.08, 1.28) | 0.0004 | 0.97 (0.88, 1.07) | 0.5397 | 0.95 (0.86, 1.05) | 0.2834 |
| **Vascular dementia** |  |  |  |  |  |  |  |  |
| MAFLD–/MASLD– | 248,213 | 602 (0.24) | Reference | - | Reference | - | Reference | - |
| MAFLD+/MASLD– | 43,355 | 170 (0.39) | 1.62 (1.37, 1.92) | <0.0001 | 1.37 (1.11, 1.70) | 0.0031 | 1.30 (1.08, 1.56) | 0.0061 |
| MAFLD–/MASLD+ | 225 | 0 (0.00) | - | - | - | - | - | - |
| MAFLD+/MASLD+ | 111,713 | 502 (0.45) | 1.86 (1.65, 2.09) | <0.0001 | 1.41 (1.23, 1.62) | <0.0001 | 1.30 (1.14, 1.48) | <0.0001 |
| **Frontotemporal dementia** |  |  |  |  |  |  |  |  |
| MAFLD–/MASLD– | 248,213 | 110 (0.04) | Reference | - | Reference | - | Reference | - |
| MAFLD+/MASLD– | 43,355 | 19 (0.04) | 0.99 (0.61, 1.61) | 0.9641 | 0.97 (0.58, 1.65) | 0.9211 | 0.97 (0.57, 1.64) | 0.9049 |
| MAFLD–/MASLD+ | 225 | 0 (0.00) | - | - | - | - | - | - |
| MAFLD+/MASLD+ | 111,713 | 51 (0.05) | 1.03 (0.74, 1.44) | 0.8609 | 0.79 (0.56, 1.11) | 0.178 | 0.78 (0.55, 1.11) | 0.1713 |

HR, hazard ratio; MAFLD, metabolic dysfunction associated fatty liver disease; MASLD, metabolic dysfunction-associated steatotic liver disease; MetALD, MASLD with greater alcohol consumption; SLD, steatotic liver diseases.

^a^ Analysis by Cox proportional hazards model.

^b^ Adjusted for age, sex, ethnicity, education, Townsend deprivation index, income levels, smoking status, alcohol intake and IPAQ activity group.

^c^ Additionally adjusted for *APOE ε4* genotypes and cardiovascular diseases.

# Supplementary Table 15. Association of MAFLD and its subtypes with Alzheimer’s (or vascular) dementia excluding participants previously or simultaneously diagnosed with vascular (or Alzheimer’s) dementia during the follow-up period.

|  | **No. of participants** | **No. of cases (%)** | **Crude model** | | **Multivariable model 1^b^** | | **Multivariable model 2^c^** | |
| --- | --- | --- | --- | --- | --- | --- | --- | --- |
|  |  |  | **HR (95% CI)** | ***P* value^a^** | **HR (95% CI)** | ***P* value^a^** | **HR (95% CI)** | ***P* value^a^** |
| **Alzheimer’s disease** |  |  |  |  |  |  |  |  |
| MAFLD– | 248,350 | 1,323 (0.53) | Reference | - | Reference | - | Reference | - |
| MAFLD+ | 154,998 | 874 (0.56) | 1.09 (1.00, 1.18) | 0.0599 | 0.94 (0.86, 1.03) | 0.1622 | 0.92 (0.84, 1.01) | 0.0638 |
| No SLD | 247,898 | 1,321 (0.53) | Reference | - | Reference | - | Reference | - |
| Non-MAFLD steatosis | 452 | 2 (0.44) | 0.85 (0.21, 3.42) | 0.8227 | 1.10 (0.27, 4.39) | 0.8963 | 1.13 (0.28, 4.51) | 0.8684 |
| MAFLD (diabetes) | 18,319 | 199 (1.09) | 2.25 (1.93, 2.61) | <.0001 | 1.49 (1.28, 1.73) | <.0001 | 1.41 (1.21, 1.64) | <.0001 |
| MAFLD (overweight/obesity) | 133,885 | 661 (0.49) | 0.94 (0.86, 1.03) | 0.2016 | 0.85 (0.77, 0.94) | 0.0009 | 0.84 (0.76, 0.92) | 0.0003 |
| MAFLD (lean metabolic disorder) | 2,794 | 14 (0.50) | 0.99 (0.58, 1.67) | 0.9668 | 0.82 (0.48, 1.39) | 0.4628 | 0.82 (0.48, 1.39) | 0.4657 |
| **Vascular dementia** |  |  |  |  |  |  |  |  |
| MAFLD– | 248,365 | 529 (0.21) | Reference | - | Reference | - | Reference | - |
| MAFLD+ | 155,012 | 616 (0.40) | 1.91 (1.70, 2.15) | <0.0001 | 1.50 (1.33, 1.69) | <0.0001 | 1.38 (1.22, 1.55) | <0.0001 |
| No SLD | 247,913 | 528 (0.21) | Reference | - | Reference | - | Reference | - |
| Non-MAFLD steatosis | 452 | 1 (0.22) | 1.07 (0.15, 7.58) | 0.9488 | 1.17 (0.16, 8.32) | 0.8759 | 1.30 (0.18, 9.26) | 0.7928 |
| MAFLD (diabetes) | 18,326 | 229 (1.25) | 6.43 (5.51, 7.51) | <0.0001 | 3.80 (3.24, 4.46) | <0.0001 | 3.09 (2.63, 3.64) | <0.0001 |
| MAFLD (overweight/obesity) | 133,891 | 370 (0.28) | 1.32 (1.15, 1.50) | <0.0001 | 1.09 (0.95, 1.25) | 0.2111 | 1.04 (0.90, 1.19) | 0.6163 |
| MAFLD (lean metabolic disorder) | 2,795 | 17 (0.61) | 3.00 (1.85, 4.86) | <0.0001 | 2.16 (1.33, 3.51) | 0.0019 | 2.17 (1.33, 3.52) | 0.0018 |

HR, hazard ratio; MAFLD, metabolic dysfunction associated fatty liver disease; MASLD, metabolic dysfunction-associated steatotic liver disease; MetALD, MASLD with greater alcohol consumption; SLD, steatotic liver diseases.

^a^ Analysis by Cox proportional hazards model.

^b^ Adjusted for age, sex, ethnicity, education, Townsend deprivation index, income levels, smoking status, alcohol intake and IPAQ activity group.

^c^ Additionally adjusted for *APOE ε4* genotypes and cardiovascular diseases.

# Supplementary Table 16. Association of MASLD and SLD subtypes with Alzheimer's (or vascular) dementia excluding participants previously or simultaneously diagnosed with vascular (or Alzheimer's) dementia during the follow-up period.

|  | **No. of participants** | **No. of cases (%)** | **Crude model** | | **Multivariable model 1^b^** | | **Multivariable model 2^c^** | |
| --- | --- | --- | --- | --- | --- | --- | --- | --- |
|  |  |  | **HR (95% CI)** | ***P* value^a^** | **HR (95% CI)** | ***P* value^a^** | **HR (95% CI)** | ***P* value^a^** |
| **Alzheimer’s disease** |  |  |  |  |  |  |  |  |
| MASLD– | 291,468 | 1,509 (0.52) | Reference | - | Reference | - | Reference | - |
| MASLD+ | 111,880 | 688 (0.61) | 1.21 (1.11, 1.33) | <0.0001 | 1.01 (0.92, 1.11) | 0.8034 | 0.99 (0.90, 1.09) | 0.8818 |
| No SLD | 247,898 | 1,321 (0.53) | Reference | - | Reference | - | Reference | - |
| MASLD | 111,880 | 688 (0.61) | 1.18 (1.08, 1.30) | 0.0004 | 0.98 (0.89, 1.08) | 0.7172 | 0.96 (0.87, 1.06) | 0.4108 |
| MetALD | 43,516 | 188 (0.43) | 0.84 (0.72, 0.97) | 0.0204 | 0.81 (0.69, 0.95) | 0.0078 | 0.79 (0.68, 0.93) | 0.0043 |
| Cryptogenic SLD | 30 | 0 (0.00) | - | - | - | - | - | - |
| Other specific etiology SLD | 24 | 0 (0.00) | - | - | - | - | - | - |
| **Vascular dementia** |  |  |  |  |  |  |  |  |
| MASLD– | 291,480 | 684 (0.23) | Reference | - | Reference | - | Reference | - |
| MASLD+ | 111,897 | 461 (0.41) | 1.79 (1.59, 2.01) | <0.0001 | 1.39 (1.24, 1.57) | <0.0001 | 1.28 (1.14, 1.45) | <0.0001 |
| No SLD | 247,913 | 528 (0.21) | Reference | - | Reference | - | Reference | - |
| MASLD | 111,897 | 461 (0.41) | 1.98 (1.75, 2.24) | <0.0001 | 1.51 (1.32, 1.71) | <0.0001 | 1.38 (1.21, 1.57) | <0.0001 |
| MetALD | 43,513 | 155 (0.36) | 1.72 (1.44, 2.06) | <0.0001 | 1.46 (1.21, 1.77) | <0.0001 | 1.38 (1.14, 1.66) | 0.0008 |
| Cryptogenic SLD | 30 | 0 (0.00) | - | - | - | - | - | - |
| Other specific etiology SLD | 24 | 1 (4.17) | 25.9 (3.64, 183.9) | 0.0012 | 40.1 (5.62, 285.9) | 0.0002 | 23.5 (3.29, 168.1) | 0.0016 |

HR, hazard ratio; MAFLD, metabolic dysfunction associated fatty liver disease; MASLD, metabolic dysfunction-associated steatotic liver disease; MetALD, MASLD with greater alcohol consumption; SLD, steatotic liver diseases.

^a^ Analysis by Cox proportional hazards model.

^b^ Adjusted for age, sex, ethnicity, education, Townsend deprivation index, income levels, smoking status, alcohol intake and IPAQ activity group.

^c^ Additionally adjusted for *APOE ε4* genotypes and cardiovascular diseases.

# Supplementary Table 17. Risk of Alzheimer's (or vascular) dementia according to presence and combination of MAFLD and/or MASLD excluding participants previously or simultaneously diagnosed with vascular (or Alzheimer's) dementia during the follow-up period.

|  | **No. of participants** | **No. of cases (%)** | **Crude model** | | **Multivariable model 1^b^** | | **Multivariable model 2^c^** | |
| --- | --- | --- | --- | --- | --- | --- | --- | --- |
|  |  |  | **HR (95% CI)** | ***P* value^a^** | **HR (95% CI)** | ***P* value^a^** | **HR (95% CI)** | ***P* value^a^** |
| **Alzheimer’s disease** |  |  |  |  |  |  |  |  |
| MAFLD–/MASLD– | 248,125 | 1,323 (0.53) | Reference | - | Reference | - | Reference | - |
| MAFLD+/MASLD– | 43,343 | 186 (0.43) | 0.83 (0.71, 0.97) | 0.0163 | 0.80 (0.68, 0.94) | 0.0057 | 0.79 (0.67, 0.92) | 0.003 |
| MAFLD–/MASLD+ | 225 | 0 (0.00) | - | - | - | - | - | - |
| MAFLD+/MASLD+ | 111,655 | 688 (0.62) | 1.18 (1.08, 1.30) | 0.0003 | 0.98 (0.89, 1.08) | 0.7228 | 0.96 (0.87, 1.06) | 0.4141 |
| **Vascular dementia** |  |  |  |  |  |  |  |  |
| MAFLD–/MASLD– | 248,140 | 529 (0.21) | Reference | - | Reference | - | Reference | - |
| MAFLD+/MASLD– | 43,340 | 155 (0.36) | 1.73 (1.44, 2.06) | <0.0001 | 1.46 (1.21, 1.77) | <0.0001 | 1.38 (1.14, 1.66) | 0.0008 |
| MAFLD–/MASLD+ | 225 | 0 (0.00) | - | - | - | - | - | - |
| MAFLD+/MASLD+ | 111,672 | 461 (0.41) | 1.98 (1.75, 2.24) | <0.0001 | 1.51 (1.32, 1.71) | <0.0001 | 1.38 (1.21, 1.57) | <0.0001 |

HR, hazard ratio; MAFLD, metabolic dysfunction associated fatty liver disease; MASLD, metabolic dysfunction-associated steatotic liver disease; MetALD, MASLD with greater alcohol consumption; SLD, steatotic liver diseases.

^a^ Analysis by Cox proportional hazards model.

^b^ Adjusted for age, sex, ethnicity, education, Townsend deprivation index, income levels, smoking status, alcohol intake and IPAQ activity group.

^c^ Additionally adjusted for *APOE ε4* genotypes and cardiovascular diseases.

# STROBE Statement—Checklist of items that should be included in reports of *cohort studies*

|  | Item No | Recommendation | Pages |
| --- | --- | --- | --- |
| **Title and abstract** | 1 | (*a*) Indicate the study’s design with a commonly used term in the title or the abstract | 1-2 |
|  |  | (*b*) Provide in the abstract an informative and balanced summary of what was done and what was found | 2-3 |
| Introduction | | |  |
| Background/rationale | 2 | Explain the scientific background and rationale for the investigation being reported | 6-7 |
| Objectives | 3 | State specific objectives, including any prespecified hypotheses | 7 |
| Methods | | |  |
| Study design | 4 | Present key elements of study design early in the paper | 8 |
| Setting | 5 | Describe the setting, locations, and relevant dates, including periods of recruitment, exposure, follow-up, and data collection | 8 |
| Participants | 6 | (*a*) Give the eligibility criteria, and the sources and methods of selection of participants. Describe methods of follow-up | 8-9 |
|  |  | (*b*) For matched studies, give matching criteria and number of exposed and unexposed | - |
| Variables | 7 | Clearly define all outcomes, exposures, predictors, potential confounders, and effect modifiers. Give diagnostic criteria, if applicable | 8-9 |
| Data sources/ measurement | 8* | For each variable of interest, give sources of data and details of methods of assessment (measurement). Describe comparability of assessment methods if there is more than one group | *9* |
| Bias | 9 | Describe any efforts to address potential sources of bias |  |
| Study size | 10 | Explain how the study size was arrived at |  |
| Quantitative variables | 11 | Explain how quantitative variables were handled in the analyses. If applicable, describe which groupings were chosen and why | 10 |
| Statistical methods | 12 | (*a*) Describe all statistical methods, including those used to control for confounding | 11 |
|  |  | (*b*) Describe any methods used to examine subgroups and interactions | 11 |
|  |  | (*c*) Explain how missing data were addressed | 10-11 |
|  |  | (*d*) If applicable, explain how loss to follow-up was addressed | NA |
|  |  | (*e*) Describe any sensitivity analyses | 11-12 |
| Results | | |  |
| Participants | 13* | (a) Report numbers of individuals at each stage of study—eg numbers potentially eligible, examined for eligibility, confirmed eligible, included in the study, completing follow-up, and analysed | 12 |
|  |  | (b) Give reasons for non-participation at each stage | 12 |
|  |  | (c) Consider use of a flow diagram | 12 |
| Descriptive data | 14* | (a) Give characteristics of study participants (eg demographic, clinical, social) and information on exposures and potential confounders | 12-13 |
|  |  | (b) Indicate number of participants with missing data for each variable of interest | 12 |
|  |  | (c) Summarise follow-up time (eg, average and total amount) | 13 |
| Outcome data | 15* | Report numbers of outcome events or summary measures over time | 13 |
| Main results | 16 | (*a*) Give unadjusted estimates and, if applicable, confounder-adjusted estimates and their precision (eg, 95% confidence interval). Make clear which confounders were adjusted for and why they were included | 13-15 |
|  |  | (*b*) Report category boundaries when continuous variables were categorized | 13-15 |
|  |  | (*c*) If relevant, consider translating estimates of relative risk into absolute risk for a meaningful time period | - |
| Other analyses | 17 | Report other analyses done—eg analyses of subgroups and interactions, and sensitivity analyses | 15-16 |
| Discussion | | |  |
| Key results | 18 | Summarise key results with reference to study objectives | 16 |
| Limitations | 19 | Discuss limitations of the study, taking into account sources of potential bias or imprecision. Discuss both direction and magnitude of any potential bias | 21 |
| Interpretation | 20 | Give a cautious overall interpretation of results considering objectives, limitations, multiplicity of analyses, results from similar studies, and other relevant evidence | 16-21 |
| Generalisability | 21 | Discuss the generalisability (external validity) of the study results | 21 |
| Other information | | |  |
| Funding | 22 | Give the source of funding and the role of the funders for the present study and, if applicable, for the original study on which the present article is based | 22 |
